# Family systems approaches in pediatric obesity management: a scoping review

**DOI:** 10.1186/s12887-024-04646-w

**Published:** 2024-04-02

**Authors:** Natasha Wills-Ibarra, Keryn Chemtob, Heather Hart, Francesca Frati, Keeley J Pratt, Geoff DC Ball, Andraea Van Hulst

**Affiliations:** 1 Ingram School of Nursing, Faculty of Medicine and Health Sciences, McGill University, 680 Sherbrooke West Suite 1800, Montreal, QC Canada; 2 Department of Human Sciences, Human Development and Family Science Program, Couple and Family Therapy Specialization, College of Education and Human Ecology, The Ohio State University, Columbus, OH USA; 3 Department of Surgery, The Ohio State University Wexner Medical Centre, Columbus, OH USA; 4 Department of Pediatrics, Faculty of Medicine & Dentistry, University of Alberta, Edmonton, AB Canada

**Keywords:** Children and adolescents, Childhood obesity, Family systems, Lifestyle behaviours, Obesity management

## Abstract

Family-based obesity management interventions targeting child, adolescent and parental lifestyle behaviour modifications have shown promising results. Further intervening on the family system may lead to greater improvements in obesity management outcomes due to the broader focus on family patterns and dynamics that shape behaviours and health. This review aimed to summarize the scope of pediatric obesity management interventions informed by family systems theory (FST). Medline, Embase, CINAHL and PsycInfo were searched for articles where FST was used to inform pediatric obesity management interventions published from January 1980 to October 2023. After removal of duplicates, 6053 records were screened to determine eligibility. Data were extracted from 50 articles which met inclusion criteria; these described 27 unique FST-informed interventions. Most interventions targeted adolescents (44%), were delivered in outpatient hospital settings (37%), and were delivered in person (81%) using group session modalities (44%). Professionals most often involved were dieticians and nutritionists (48%). We identified 11 FST-related concepts that guided intervention components, including parenting skills, family communication, and social/family support. Among included studies, 33 reported intervention effects on at least one outcome, including body mass index (BMI) (*n* = 24), lifestyle behaviours (physical activity, diet, and sedentary behaviours) (*n* = 18), mental health (*n* = 12), FST-related outcomes (*n* = 10), and other outcomes (e.g., adiposity, cardiometabolic health) (*n* = 18). BMI generally improved following interventions, however studies relied on a variety of comparison groups to evaluate intervention effects. This scoping review synthesises the characteristics and breadth of existing FST-informed pediatric obesity management interventions and provides considerations for future practice and research.

**Supplementary Information:**

The online version contains supplementary material available at 10.1186/s12887-024-04646-w.

## Background

Obesity is a major public health concern affecting all age groups [1]. The high global prevalence of childhood overweight and obesity is concerning given known impacts on several body systems, including the cardiovascular, pulmonary, endocrine, gastrointestinal and musculoskeletal systems [2]. Obesity persists from childhood into adulthood [3] resulting in increased risk of morbidity and mortality [4, 5]. In addition to its bearings on physical health, childhood overweight and obesity are associated with poor psychosocial outcomes [2, 6]. Given its multiple immediate and long-term consequences, managing overweight and obesity in children and adolescents through effective interventions is a priority.

Most pediatric obesity management interventions fall within the umbrella of family-based approaches, targeting specific lifestyle behaviours (e.g., diet, physical activity) for obesity management and including at least one family member (e.g., a parent) in addition to the target child. Family-based behavioural interventions have shown improvements in lifestyle behaviours and in obesity-related outcomes [7–10]. However, these interventions may have limited effects if they fail to address the family patterns and dynamics that shape lifestyle behaviours [11].

Family Systems Theory (FST) has gained attention in pediatric obesity management [12]. Derived from general systems theory, FST focuses on understanding the interrelationships between elements within a system (e.g., the dynamics of a family unit, communication, and problem-solving). It views families as complex systems in which events or changes in one family member influence other interrelated parts of the system [11]. FST explicitly recognizes the key roles of family-level influences on children’s lifestyle behaviours and changes therein, with the goal of promoting health and managing obesity [13]. The integration of a family systems approach in pediatric obesity management interventions may increase their efficacy and sustainability by targeting core family dynamics that challenge lifestyle modifications required for obesity management [12]. A preliminary search of published systematic reviews on family-based obesity management interventions revealed a limited focus on family systems approaches with few reviews identifying specific intervention components consistent with FST [10, 14–18]. Family systems concepts (e.g., interpersonal dynamics, family functioning, family problem-solving) were infrequently mentioned or only discussed narrowly [12]. Moreover, despite the potential benefits of using FST, clinicians have reported a lack of clarity regarding how to apply FST in the context of pediatric obesity management [13].

This scoping review addresses the following overarching question: How has FST been used in the context of pediatric obesity management interventions? Specifically, this review identifies 1) who is targeted by existing FST-informed interventions; 2) settings where they have been implemented (primary, specialty/tertiary, community); 3) delivery format (e.g., group vs. individual, parents-only vs. child-only vs. family) and professionals involved in the implementation of these interventions; 4) FST-related concepts that are integrated into interventions and tools used to measure these concepts; and 5) effects of FST-informed approaches on obesity outcomes and on FST-related concepts.

## Methods

A scoping review of the literature was conducted following the Joanna Briggs Institute (JBI) methodology [19], and the PRISMA-ScR and PRISMA-S guidelines for searches [20, 21].

### Search strategy

A comprehensive search strategy was used. An academic health sciences librarian (FF) conducted a preliminary search that allowed us to analyse titles, abstracts, and index terms of isolated papers in order to refine our scoping review questions and define the final search strategy. Although we initially wanted to use a broad approach to the definition of FST, for feasibility reasons, we narrowed our review to articles that explicitly mention the use of FST to inform the development of obesity management interventions [12]. Similarly, although we initially wanted to include both prevention and management interventions, we narrowed our review to interventions focusing on obesity management (i.e., children and adolescents with overweight or obesity). Following these refinements, a final search strategy was developed by FF and a peer review of the search strategy was conducted by a second academic health sciences librarian using the PRESS (Peer Review of Electronic Search Strategies) guideline [22]. After minor revisions, the final search was run in Medline, Cumulative Index to Nursing and Allied Health Literature (CINAHL) Embase, and PsycInfo on April 4, 2020. Duplicates across databases were removed in EndNote using a simplified method described by Bramer et al. [23] and additional duplicates were identified in Rayyan [24]. Our search was based on three main concepts, namely family systems, pediatric obesity, and interventions. The full search strategies for all four databases are presented in Supplemental Table 1. We also examined reference lists and citations of included studies for further pertinent studies that were not captured through our database searches. This overall search strategy was implemented for studies published between January 1980 and April 2020. No additional limits or search filters were used. In October 2023, we updated our review by conducting the same search in Medline to identify publications indexed between April 4, 2020 and October 27, 2023, the date of this search. We also searched for articles published in the last 3 years that cited previously identified research protocol articles of FST-informed obesity management interventions. This scoping review thus includes articles published between January 1980 and October 2023; this date range was selected to capture early family systems interventions following the increased recognition by the early 1990’s of the role of families in childhood obesity [25].

### Inclusion and exclusion criteria

Details regarding inclusion and exclusion criteria are presented in Table 1. Articles that used FST to inform the design of a pediatric obesity management intervention or program were included. Specifically, we included publications describing obesity management interventions that focus on children aged 2 to 18 years, with overweight or obesity, the direct involvement of at least one adult family member, and the explicit statement of a family systems-related theory, model, and/or framework [12]. Review papers, case studies, texts, opinion papers, letters and gray literature were excluded.

**Table 1:** Inclusion and exclusion criteria

	**Inclusion Criteria**	**Exclusion Criteria**
**Concept**	- Explicitly mentions the use of FST to inform the design and development of a pediatric obesity management intervention: - Family theories included in our search strategy were those identified by Skelton et al. [12] in their review of family theories utilized in childhood obesity research, namely FST, Circumplex Model of Family Functioning, Double ABCX Model of Family Stress, Family Stress Model of Economic Strain, Family Development Theory, and Ecologic Systems Theory - Additional family theories included are: General Systems Theory, Calgary Family Assessment / Intervention Model, Systemic Family Therapy	- No explicit mention of FST or related theory in the design and development of the pediatric obesity management intervention - No direct involvement of family members (e.g., school-based intervention with no or minimal family involvement)
**Participants**	- Children and adolescents of both sexes, between the ages of 2–18 years - Children and adolescents with overweight or obesity as per the definition in original articles - Targets at least one adult family member with or without the identified child/adolescent with overweight/obesity	- Children less than 2 years of age - Children and adolescents without overweight or obesity (e.g., prevention interventions) - No direct involvement of family members
**Context**	- Research conducted in any country or healthcare system, in any setting where healthcare may be delivered (e.g., inpatient and outpatient clinics, the community, home-based settings, etc.) - Publications that dated between January 1980 and October 2023 - All socioeconomic status and sociocultural factors were considered	
**Types of Sources**	- Primary research articles published in peer-reviewed journals - Any language - Quantitative, qualitative and mixed methods designs - Published study protocols	- Case studies - Opinion papers - Letters - Gray literature

### Study selection

EndNote (Thomson Reuters, New York, USA) was used to manage records identified from the literature search. Search results from all databases were combined, and duplicates were removed. Records were then imported into Rayyan [26] to manage decisions on inclusion/exclusion. For the updated search covering the period of April 2020 to October 2023, we used Covidence, a web-based collaboration software platform to manage the flow of records in review studies. Titles and abstracts were screened for inclusion by two out of four independent reviewers (NWI, KC and 2 research assistants), followed by screening of full-text by two of the same reviewers. Discordances at both stages were settled by the senior author (AVH).

### Data extraction, analysis and synthesis

Data extraction, analysis and synthesis were conducted by two reviewers (NWI, KC) and verified by the senior author. An adaptation of the JBI data extraction instrument was used to import data into a table with the following fields based on the research questions: country and name of intervention; sample size (if applicable); study design; target population (e.g., age/sex of child, family members targeted, racial/ethnic groups, etc.); type of care setting (e.g., community, hospital); description and duration of the intervention; delivery format of the intervention (e.g., group vs. individual, parents-only vs. child/teen-only vs. family); professionals involved in the intervention; Family Systems related theory or framework and other theories used to inform the intervention; specific Family Systems concepts used (e.g., family dynamics, family functioning, parenting styles, etc.); and measurment of family concepts. The results of articles that reported intervention effects on outcomes were summarized in a separate table, including intervention effects on family systems concepts, mental health, lifestyle behaviours, body mass index (BMI) and other outcomes examined. The type of control group was classified as not applicable (no control group), waitlist control, usual care, or intervention control group, with descriptors provided when available. Intervention effects were summarised based on whether an improvement, a deterioration, or the absence of changes on outcomes were reported. No standardised metrics for outcomes were sought given the diversity of included studies.

All data extracted from articles were compiled using counts and proportions to answer our research questions. A conventional inductive content analysis was completed [27] in order to identify and summarize the FST-related concepts that were intervened upon in included studies. To do so, keywords and descriptive texts were extracted from the studies’ intervention descriptions and grouped into categories with similar content; once complete, these categories were individually labelled to represent different FST-related concepts.

## Results

**Fig.1:**
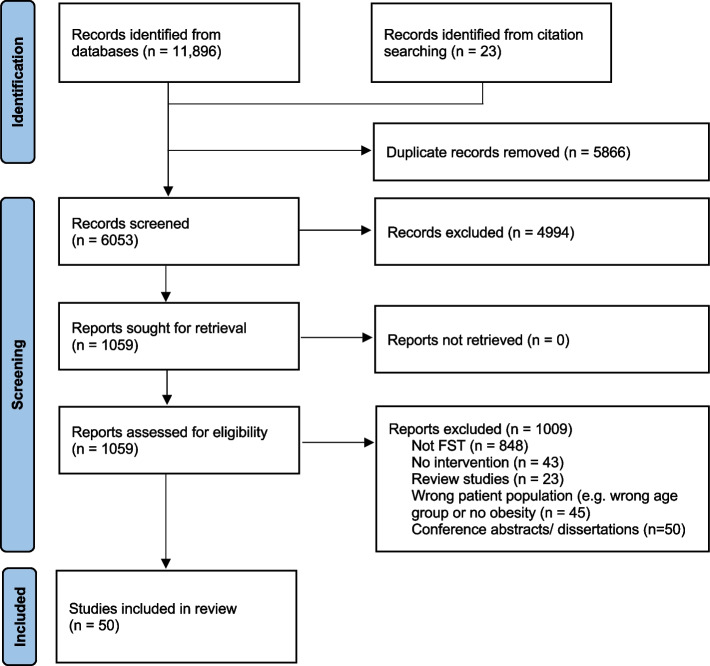
PRISMA flow diagram

Database and citation searches allowed us to identify 6053 records after the removal of duplicates, with a total of 50 articles that met inclusion criteria (Fig. 1). The most common reasons for exclusion were the absence of FST-related theory in the development of the intervention, and interventions not focusing specifically on children/adolescents with overweight/obesity. Among the included studies, all were published in English, 14 were descriptive articles (e.g., study protocols), 33 reported on at least one measured intervention outcome, 3 used qualitative post-intervention exploratory designs, and one included baseline data only. Supplemental Table 2 provides a summary of the 50 studies included in this review. Among included studies, we identified 27 unique FST-informed interventions which are presented in Table 2.

**Table 2:** Description of the obesity management interventions included in the review (*n* = 27)

**Intervention / Program Name**	**Child Age Group**^a^	**Intervention Target**	**Sample Characteristics**	**Country**	**Type of Care Setting**	**Duration of Intervention**	**Group vs. Individual Delivery**	**Delivery Focus**	**In-person vs. Online**	**Professionals Involved**
**Creating Health Environments for Chicago Kids (CHECK) Trial** [28]	SA	Parent/guardian & child	Low-income families	USA	Recruitment in Outpatient Clinic, Home-delivered	12 months	Individual	Family	In-person & telephone	Dietitians Nurses Exercise physiologist Social worker
**Dyad plus** [29]	AD	Parent/guardian & adolescent	Recruitment site characteristics: 58% female adolescents; 45% White, 32% African American, 18% Hispanic, 5% other	USA	Weight loss clinic	24 months	Group & Individual	Family	In-person	Medical providers Dietitians Behavioralists Exercise specialists
**ENTREN-F** [30, 31]	SA	Parent/guardian & child	42.7% girls; 36% low SES, 32% medium SES, 36% high SES	Spain	Outpatient clinic	6 months	Group & Individual	Family	In-person	Psychologists Psychiatrist Dietitian- nutritionist Dietary coach Physical activity experts Pediatricians Nurses
**Exergaming for Health** [32]	SA	Parent/guardian & child	Neighborhoods with poverty; 54% girls; 61% White, 25% Black, 8% Hispanic/Asian	USA	Community	6 months	Group	Family	In-person	Dietician Licensed counselor Medical students
**Familias Unidas (United Families for Health and Wellness-FUHW)** [33, 34]	AD	Parent/guardian & adolescent	Hispanic families living in USA; 52.3% females, Income in USD: 62% < 30K, 20% 30K–50K, 9%> 50K	USA	Community	3 months	Group & Individual	Family, Parent-only & AD-only	In-person	Bilingual park coaches and fitness instructors (trained on problem-posing and participatory learning)
**Families Improving Together (FIT)** [35–41]	AD	Parent/guardian & adolescent	African American families 64% female adolescents Parent annual income in USD: 3% unreported, 31% < 24K, 55% 25K–69K, 10% > 70K	USA	Community	6 months [35–39] 8 weeks [40]	Group & Individual	Family & Parent-only	In-person & online	Trained facilitators (background not specified)
**Families Improving Together- Telehealth (FIT-T)** [42]	AD	Parent/guardian & adolescent	Families of diverse backgrounds and identities	USA	Outpatient clinic	3 days of intensive behavioral intervention + telephonic wellness follow-up (duration not reported)	Individual	Families	In-person & Teleconference	Psychologists Licensed clinicians A postdoctoral fellow with interest in health promotion and program development
**Families on the Move (FOTM)** [43]	SA	Mother & child	Latino families. 57.9% girls Maternal education: 71% Less than high school, 14% high school diploma or general education diploma, 14% college graduate or trade school	USA	Community	2 months	Group	Family & Parent-only & Child-only	In-person	Pediatric nurse practitioner student Primary investigator
**Family Connections** [44–46]	SA	Parent/guardian	Families in a medically underserved region 58% girls 45% black, 48% white, 8% other; 9% Hispanic Parental income in USD: 29% < 20K, 47% 20K–55K, 24% > 55K	USA	Community	6 months [45]; 12 months [44]	Group & Individual	Parent-only	In-person & online	Dietician Local Parks and Recreation staff
**Family Weight School Model** [47]	AD	Parent/guardian & adolescent	50% female adolescents	Sweden	Obesity center	12 months	Group & Individual	Family	In-person	Pediatrician Dietician / sports trainer Pediatric nurse Family therapist
**Fit Kids / Fit Families (FKFF)** [48]	SA AD	Parent/guardian & child/adolescent	66% female adolescents	USA	Community	3 months	Group	Family & Parent-only & Child-only	In-person	Nurse Dietician Behaviourist Exercise specialist
**Diabetes Prevention Program among Latino Youths** [49, 50]	AD	Parent/guardian & adolescent	Latino families, adolescents with prediabetes, 40.1% female adolescents	USA	Community	9 months 6 months [50]	Group	Family & Child-only	In-person	Trained health educators Trained Physical Activity instructors
**Lighter Living program (LiLi)** [51]	PS	Parent/guardian	50% girls; 93% native Swedish, 3% European, 3% South American. Parental education level: 10% compulsory school, 60% high school, 40% college/ university	Sweden	Hospital (outpatient)	12 months	Group	Parent-only	In-person	Therapists Occupational therapists
**Lund Overweight and Obesity Preschool Study (LOOPS)** [52]	PS	Parent/guardian	N/A	Sweden	Hospital (outpatient)	12 months	Group	Parent-only	In-person	Clinical psychologist Occupational therapists
**Mind, Exercise, Nutrition, Do it! (MEND)** [53–55]	PS SA	Parent/guardian & child	Hispanic and Black families, 50% girls, 88% Hispanic, [53] 54% females, 50% white, social class (by occupation): 39% non-manual [54, 55]	USA [53] UK [54, 55]	Community [53, 55] Hospital (outpatient) [54]	12 months [53, 54]; 2 months [55]	Group [53, 55] Group & Individual [54]	Family	In-person	Healthcare providers MEND leaders and assistants
**Motivational plus family weight loss intervention** [56]	AD	Parent/guardian & adolescent	Low income families, 70% female adolescents, 65% African American, 35% Caucasian	USA	Community	1.5 months	Group	Family & Parent-only & AD-only	In-person	Not specified
**Multidisciplinary family-based behavioral therapy for obesity (FBBT)** [57]	AD	Parent/guardian & adolescent	62% female adolescents	Switzerland	Hospital (outpatient)	5 months	Group	Family& Parent-only & AD-only	In-person	Licensed counselors
**Multidisciplinary Treatment Program** [58, 59]	PS	Parent/guardian & child	72.15% girls	Netherlands	Hospital (outpatient)	4 months	Group & Individual	Family & Parent-only	In-person	Dietician Physiotherapist Psychologist
**Multifamily Therapy plus Psychoeducation** [60]	AD	Parent/guardian & adolescent	Female adolescents	USA	Hospital (outpatient)	4 months	Group	Family	In-person	Family therapists (master-level) Trained graduate students
**Multisystemic Therapy** [61–63]	AD	Parent/guardian & adolescent	Low-income, African American adolescents, 77% female adolescents	USA	Community & Home	6.5 months	Individual	Family	In-person	Therapists
**Intervention not named** [64]	SA	Parent/guardian & child	62% girls	Sweden	Outpatient clinic	16 months	Individual	Family	In-person	Nutritionists Dieticians Psychologists Pediatricians
**Parents as Agents of Change (PAC)** [65–68]	SA	Parent/guardian	52% girls; 73.1% white	Canada	Hospital (outpatient) [65, 67, 68]; Community [66]	4 months	Group	Parent-only	In-person	Nutritionists Psychologists Social Workers Physiotherapists Trained on Cognitive Behavioral Therapy [68]
**Positively Fit** [69]	SA AD	Parent/guardian & child/adolescent	59.1% female children and adolescents; 14% African American, 71% European American, 4% Latino, 4% Biracial, 7% Other. Mean monthly income: $4072.54 USD	USA	Hospital (outpatient)	2.5 months	Group	Family	In-person	Nutritionists Therapists
**SHINE** [70, 71]	AD	Parent/guardian & adolescent	African American families, 60% female adolescents. Yearly family income in USD: 33% < 24K, 44% 25K–54 (44%), > 55K	USA	Community	1.5 months	Group [71] Group & Individual [70]	Family	In-person	Trained graduate students (in Psychology or Public Health)
**Solution-focused family therapy** [72]	SA AD	Parent/guardian & child/adolescent	40% female children and adolescents	Sweden	Hospital (outpatient)	15 months	Individual	Family	In-person	Dietician Sports Trainer Pediatric nurse Family Therapist
**Standard Behavioral Treatment + Enhanced Parenting** [73, 74]	AD	Parent/guardian & adolescent	76% female adolescents, 67.5% non-Hispanic whites. Parental education: 82% college or more	USA	Hospital (outpatient)	4 months	Group	Parent-only & AD-only & Family	In-person	Psychologists Nutritionists Clinical psychology graduate students Bachelor-level research staffs
**T.A.F.F. (Telephone based Adiposity prevention For Families)** [75–77]	SA AD	Parent/guardian & child/adolescent	59% female children and adolescents	Germany	Community	12 months	Individual	Parent-only & AD-only & Family	Telephone-based	Prevention managers / counsellors

^a^*PS* Preschool, *SA* School-age, *AD* Adolescent

### Who is targeted by existing FST-informed interventions?

Of the 27 unique interventions, 3 (11%) targeted preschool children exclusively, 7 (26%) targeted school-aged children exclusively, and 12 (44%) targeted adolescents exclusively. In addition, one intervention (4%) targeted both preschool and school-aged children, while 4 (15%) targeted both school-aged children and adolescents. Twenty-three interventions (85%) targeted the child/adolescent and at least one parent/guardian, and the remaining 4 interventions (15%) targeted a parent/guardian without the index child/adolescent. Five interventions (19%) were designed for families with low incomes or living in underserved areas. Some interventions targeted specific ethnic or population sub-groups, including 4 interventions (15%) for African American families, 3 (11%) for Latin American families, one for Hispanic and Black families, and one for female adolescents only.

### In which settings are FST-informed approaches implemented?

All studies were conducted in Western countries, including the USA, Europe, and Canada. Four different intervention settings were identified: outpatient hospital (37%, *n* = 10), community-based (26%, *n* = 7), pediatric obesity management center (7%, *n* = 2), and home-based (7%, *n* = 2). An additional 6 interventions (22%) relied on a combination of settings, 4 of which included a home-based component (15%).

### How are FST-informed interventions delivered, and which professionals are involved?

Intervention duration ranged from 1.5 to 24 months (median of 6 months). Most interventions were delivered entirely in person (81%, *n* = 22). Three interventions (11%) used a combination of in-person and virtual/online sessions, one intervention combined in-person and telephone delivery, and one intervention was delivered entirely over the phone. Twelve interventions (44%) were group-based, 6 (22%) were delivered individually, and 9 (33%) used a combination of group and individual sessions.

In terms of in-session participation, 12 interventions (44%) comprised sessions that included the child/adolescent together with at least one adult family member at all times, whereas another 11 (41%) had a mix of parent-only, child/adolescent-only, and parent–child/adolescent sessions. The remaining 4 interventions (15%) included only parents in their intervention, without the child/adolescent.

Interventions were delivered by a wide range of health professionals, and commonly involved two or more professionals. These included dieticians/nutritionists (48%, *n* = 13), licensed counsellors/therapists (30%, *n* = 8), psychologists (30%, *n* = 8), sports trainers and exercise specialists (30%, *n* = 8), students in different health-related fields (22%, *n* = 6), nurses (19%, *n* = 5), pediatricians (15%, *n* = 4), occupational therapists (7%, *n* = 2), physiotherapists (7%, *n* = 2), social workers (7%, *n* = 2), health educators (4%, *n* = 1), and behaviouralists (4%, *n* = 1). Moreover, 7 of the interventions (26%) included other non-health-related professionals (e.g., local parks and recreation staff, prevention managers, and trained facilitators with unspecified backgrounds), or did not specify the professionals involved.

### Which FST-related concepts are included in interventions and how are these concepts measured?

A detailed description of the 11 FST-related concepts identified across interventions, including definitions and examples of how they were integrated within interventions, is presented in Table 3. The most common concepts related to parenting skills (59%, *n* = 16), family communication (52%, *n* = 14), and social/family support (48%, *n* = 13). Other concepts included family functioning (37%, *n* = 10), parental role modelling (30%, *n* = 8), autonomy support (22%, *n* = 6), shared decision-making (19%, *n* = 5), home environment (22%, *n* = 6), empowerment (11%, *n* = 3), family goal setting (26%, *n* = 7), and family problem solving (22%, *n* = 6). Some studies reported in-depth descriptions of how FST-related concepts were integrated while others did not. Few studies included pre- or post-intervention measurements of FST-related concepts as shown in Table 3.

**Table 3:** Family systems theory-related concepts and measurement tools included in interventions (*n* = 27)

FST concept and definition^a^	Nb of interventions with concept included	Examples	References	Tools used to measure the FST concept (if applicable)
**Parenting Skills** Skills and strategies that can be useful to parents who are supporting a child in a obesity-management intervention. Effective parenting skills may vary based on existing dynamics within the family but may include limit-setting, active listening and communication, autonomy-support, parental role-modeling, etc.	16	**Families Improving Together (FIT)**: Focused on improving parenting skills around communication, autonomy support, and social support [36] **Families on the Move (FOTM)**: Focused on limit setting, re-framing the problem, re-framing parent role and child responsibility, exercising parental leadership, exercising parental general skills, promoting parent–child effective communication, promoting problem-solving skills, increasing self-efficacy in parental role of providing a positive family environment [43]	[28–30, 32–41, 43–47, 52, 53, 56, 61–63, 65–68, 70–72, 75–77]	The Parenting Strategies for Eating and Activity Scale (PEAS) [53] The Parenting Dimensions Inventory (PDI-S) [56] The Parenting Stress Index [67, 68] The Authoritative Parent Index [39] The Child Feeding Questionnaire [39, 41] Newest Vital Sign [46] Parenting practices scale [33]
**Family Communication** Strengthening both verbal and non-verbal communication among family members to create a supportive environment within the home. Communication strategies (e.g., active listening, openness, respect) can enhance emotional connections, foster productive discussions that can help in problem-solving and decision-making, and allow individuals to feel heard and validated within the family unit	14	**Families Improving Together (FIT)**: Targeted family communication strategies, including active listening, using push–pull language, and problem solving [35]	[29–40, 42–47, 60, 65–68, 70–74]	The Dyadic Communication Scale (DOCS) [73] The Family Interactions Topics questionnaire [73] The Family Relations Scale [33]
**Social / Family Support** Encouragement and support from the family and the broader social context to help a child/adolescent succeed in an obesity-management intervention. Support may include emotional, motivational, physical, financial and types of support and resources, as well as fostering a sense of community and solidarity for the individual	13	**Families Improving Together (FIT)**: Aimed to foster social support within families through take-home bonding activities, and between families through group activities [35, 37] **Multifamily Therapy plus Psychoeducation**: Promoted enlisting social support; e.g., determining the type of support needed, who can provide it, and how to ask for it [60] **SHINE**: Emphasized the importance of peer relationships during adolescence and the role parents play in managing peer relationships and healthy lifestyle behaviours. Adolescents were encourage to bring a friend to [one] session, and friends were integrated into the activities [71]	[29, 33, 35–40, 42, 43, 47, 49, 50, 56, 60–63, 70–72]	The Youth Quality of Life (YQOL) Inventory, including a social relationship subscale [49] The Support for Exercise Scale (revised version) [56] The Social Support for Eating Habits and Exercise Scale [61] Parent Relationship with Peer Group Scale [33]
**Family Functioning** Family member roles and interactions that affect day-to-day living within the home environment, including acceptance and understanding of one another, family decision-making and problem-solving processes, and general communication among family members. Simply described as the overall healthiness of a family unit	10	**Multidisciplinary FBBT**: Nutrition-related topics and systemic interventions to facilitate family functioning by reinforcing family resources and improving the emotional climate for adolescents with obesity [57]	[30, 31, 33, 35–40, 47, 56, 57, 60, 64, 70–72]	The Self-Report Family Inventory (SFI), including the conflict resolution, cohesion, and family nurturance subscales [60] The Family Adaptability and Cohesion Evaluation Scales IV (FACES IV) [67, 68] The Family Climate Scale [72] Family Questionnaire (FQ) [31] Family Assessment Device General Functioning [29]
**Parental Role Modelling** The ability of a parent to act as a role model and model healthy lifestyle behaviours through their actions. Recognizing parents as agents of change for their child’s habits and behaviours	8	**Families on the Move (FOTM)**: The intervention emphasizes parents as change agents who role model behavioural change by setting goals themselves [43] **Family Connections**: Parents provide an example of healthy lifestyle behaviours for their child, and model enjoyment of healthy foods and physical activity [44]	[30, 32, 43–46, 53, 58, 59, 61–63, 73, 74]	The Comprehensive Feeding Practices Questionnaire [53] The Weight Control Strategies Scale (WCSS) [74] Family Experiences Related to Food Questionnaire (FERFQ) [74] Spanish version of the Home Environment Survey-Physical Activity (HES-S) [30]
**Autonomy Support** Creating a family environment that fosters autonomy specific to health behaviours, with the goal of building intrinsic motivation for sustainable lifestyle changes (e.g., encouraging the child/adolescent to provide input, problem-solve, negotiate, participate in shared decision-making, and self-monitor their health behaviours)	6	**SHINE:** Targeted autonomy-supportive communication within the family and parental monitoring specific to activity and dietary behaviours. Intervention curriculum included tools for self-monitoring, and encourages adolescents to monitor their chosen target behaviours with weekly check-ins with their families [71] **Families Improving Together (FIT):** Intervention facilitators create a climate which fosters autonomy, competence, and belongingness. Adolescents have choices and are provided with opportunities to give input. Parents seek input from adolescents and negotiate rules and behaviour changes together [35]	[35–40, 42, 48, 56, 61–63, 70, 71]	None mentioned
**Shared Decision-Making** Encouraging collaboration when making decisions, particularly those surrounding health behaviours and activities such as meal planning, physical activity preferences, etc.	5	**Family Connections**: The intervention promoted the involvement of children in decision making for enjoyable physical activity [44]	[35–40, 44–46, 56, 60, 70, 71]	None mentioned
**Home Environment** Addressing barriers to healthy living that exist within the physical home environment	6	**Multidisciplinary Treatment Program**: Focused on removing unhealthy food triggers from the home environment [58] **Family Connections**: Provided strategies to restructure the home environment to support healthy food and activity options, while reducing options for unhealthy choices [44]	[28, 30, 44–46, 58, 59, 61–63, 73, 74]	Home Monitoring Checklist [28] Confusion, Hubbub, and Order Scale [28]
**Empowerment** Providing adequate tools, resources, support, and information to enhance an individual’s confidence surrounding certain tasks or behaviours and helping them achieve a sense of autonomy and self-efficacy to control a given aspect of their life	3	**LiLi**: Empowered parents with the knowledge they need to be able to suggest strategies and set meaningful goals for the family [51] **Multisystemic Therapy**: Empowered caregivers with the skills and resources to address difficulties inherent in raising adolescents, and empowered adolescents to cope with family, school, and neighborhood problems [61]	[42, 43, 51, 61–63]	None mentioned
**Family Goal setting** Working together to set goals that are important to the family unit as a whole, while taking into consideration things that are important to each individual within the family	7	**Family Connections**: Parents were trained to lead their family through regular goal setting related to physical activity and eating. They learned the process of goal setting using the 5As (assess, advise, ask, assist, arrange), learned how to keep objectives clear, and created a family action plan [44] **SHINE**: Families worked on target health behaviours in the order of their choice by setting goals, self-monitoring, and receiving weekly feedback [71]	[28–31, 42–46, 70, 71]	None mentioned
**Family Problem-Solving** Collaboration between a child/adolescent and their parent to identify and resolve a problem	6	**SHINE**: Families learned strategies for effective problem solving (e.g., defining the problem, brainstorming all possible solutions, making a joint decision, and discussing a plan for follow through) [71]	[28, 29, 31, 35–40, 42, 70, 71]	None mentioned

^a^Definitions for each Family Systems Theory concept are informed by the definitions provided by studies which included the concept

### What are the effects of FST-informed interventions?

Of the 50 articles reviewed, 33 reported on at least one intervention outcome, including BMI or BMI z-scores (*n* = 24), lifestyle behaviours (physical activity, diet, and sedentary behaviours) (*n* = 18), mental health (*n* = 12), FST-related outcomes (*n* = 10), and other outcomes (e.g., waist circumference, heart rate, blood pressure, cardiovascular fitness) (*n* = 18) (Table 4).

**Table 4:** Outcome results for interventions that included an evaluative component (*n* = 33)

**FST-informed Interventions **	**References **	**Comparison Group(s)**	**FST Outcomes**	**Mental Health Outcomes**	**BMI / zBMI Outcomes**	**Physical Activity Outcomes**	**Sedentary Behaviour Outcomes**	**Diet Outcomes**	**Other Outcomes**
**ENTREN-F**	Rojo, 2022 [31]	- CI (CBT) - CI (Behav. monitoring)	n/a	n/a	n/a	n/a	n/a	n/a	Attendance rate
**Exergaming for Health**	Christison, 2016 [32]	- UC (Classroom curriculum)	n/a	Self-esteem = (vs. BL) Self-worth + (vs. BL)	= (vs. UC)	= (vs. UC)	= (vs. UC)	= (vs. BL)	Blood pressure, heart rate, cardio-vascular fitness
**Familias Unidas (United Families for Health and Wellness-FUHW)**	Prado, 2020 [33]	- UC (Community practice)	Family communication + (vs. UC) Parent in-volvement + (vs. UC)	n/a	= (vs. UC)	= (vs. UC)	n/a	= (vs. BL)	Parental BMI and parental diet
	Perrino, 2022 [34]	- UC (Community practice)	n/a	n/a	n/a	n/a	+ (vs. BL)	n/a	n/a
**Family Connections (FC)**	Estabrooks, 2009 [44]^a^	- CI (Group based) - CI (workbook) - CI (phone based)^a^	n/a	Eating disorder behavior = (vs. BL) for all 3 intervention groups	+ (vs. BL) for all 3 intervention groups	+ (vs. BL) only for phone based CI	n/a	= (vs. BL) for all 3 intervention groups	n/a
	Zoellner, 2022 [46]	- CI (Behavioral modification)	n/a	QOL= (vs. BL and CI)	= (vs. BL and CI)	= (vs. BL and CI)	n/a	= (vs. BL and CI)	Engagement in intervention, BP (child and parent), waist circumference (parent)
**Family Weight School Model**	Nowicka, 2008 [47]	- WLC	n/a	n/a	+ (vs. WLC)	n/a	n/a	n/a	n/a
**Fit Kids / Fit Families (FKFF)**	Joosse, 2008 [48]	n/a	n/a	Self-esteem + (vs. BL)	+ (vs. BL)	+ (vs. BL)	+ (vs. BL)	n/a	Body circumference
**Lighter Living Program (LiLi)**	Orban, 2014 [51]	n/a	n/a	n/a	= (vs. BL)	n/a	n/a	n/a	n/a
**Mind, Exercise, Nutrition, Do it! (MEND)**	Law, 2014 [54]	n/a	n/a	Self-esteem + (vs. BL)	+ (vs. BL)	n/a	n/a	n/a	n/a
	Sacher, 2010 [55]	- WLC	n/a	Self-esteem + (vs. WLC)	+ (vs. WLC)	+ (vs. WLC)	+ (vs. WLC)	n/a	Waist circumference, BP, heart rate
	Wilson, 2019 [53]	n/a	+ (vs. BL)	n/a	n/a	n/a	n/a	+ (vs. BL)	n/a
**Motivational + Family Weight loss Intervention (M+FWL)**	Kitzman-Ulrich, 2011 [56]	- UC (Health education)	= (vs. BL)	n/a	+ (vs. UC)	+ (vs. UC)	n/a	+ (vs. UC)	n/a
**Multi-disciplinary Treatment Program**	Bocca, 2014 [58]	- UC (Health education and pediatrician follow up)	n/a	Health-related QOL + (vs. UC) Mental health - (vs. UC and BL)	+ (vs. UC)	+ (vs. UC)	n/a	n/a	Waist circumference, % of body fat
	Bocca, 2018 [59]	- UC (Health education and pediatrician follow up)	n/a	n/a	+ (vs. UC)	n/a	n/a	= (vs. UC)	n/a
**Multifamily Therapy + Psycho-education**	Kitzman-Ulrich, 2009 [60]	- CI (Psycho-education) -WLC	Conflict - (vs. CI and WLC)	n/a	= (vs. CI and BL)	n/a	n/a	- (vs. CI and BL)	n/a
**Multi-systemic Therapy**	Naar-King, 2009 [62]	- CI (Group weight-loss intervention)	n/a	n/a	+ (vs. CI)	n/a	n/a	n/a	% overweight, % body fat
	Ellis, 2010 [61]	- CI (Group weight-loss intervention)	+ (vs. CI)	n/a	+ (vs. CI)	n/a	n/a	+ (vs. CI)	% overweight, % body fat
**No Name**	Flodmark, 1993 [64]	- UC (Dietary counseling)	n/a	n/a	+ (vs. UC)	+ (vs. UC)	n/a	n/a	Skinfold thickness
**Parents as Agents of Change (PAC)**	Spence, 2017 [65]	n/a	n/a	n/a	n/a	n/a	n/a	n/a	Improved retention in program
	Spence, 2023 [68]	- CI (Psycho-education)	Functioning of family system= (vs. CI at 4, 10, and 16 mths)	n/a	= (vs. CI at 4, 10, and 16 months)	= (vs. CI at 4, 10, and 16 mths)	Screen time = (vs. CI at 4, 10, and 16 mths	= (vs. CI at 4, 10, and 16 mths)	Sleep, and parental stress
**Positively Fit**	Steele, 2011 [69]	n/a	n/a	Health- related QOL + (vs. BL)	+ (vs. BL)	n/a	n/a	n/a	n/a
**SHINE (Supporting Health Interactively through Nutrition and Exercise)**	St George, 2013 [71]	- CI (Health education)	+ (vs. CI)	n/a	n/a	+ (vs. CI and BL)	n/a	n/a	n/a
	St George, 2018 [70]	n/a	n/a	n/a	n/a	n/a	n/a	n/a	Parental PA
**Solution-focused Family Therapy**	Nowicka, 2007 [72]	n/a	+ (vs. BL)	Self-esteem + (vs. BL)	+ (vs. BL)	n/a	n/a	n/a	n/a
**Standard Behavioral Treatment + Enhanced Parenting (SBT+EP)**	Hadley, 2015 [73]	- CI (Behavioral modification)	= (vs. CI)	n/a	+ (vs. BL)	n/a	n/a	n/a	n/a
	Jelalian, 2015 [74]	- CI (Behavioral modification)	- (vs. CI)	n/a	- (vs. CI)	n/a	n/a	n/a	n/a
**T.A.F.F. (Telephone-based adiposity prevention for Families)**	Herget, 2015 [75]	n/a	n/a	Body dissatisfaction & self-efficacy + (vs. BL)	n/a	n/a	n/a	n/a	n/a
	Markert, 2014 [76]	- No details on control group	n/a	Health-related QOL + (vs. BL)	+ (vs. control)	= (vs. BL)	= (vs. BL)	= (vs. BL)	n/a
**FIT (Families Improving Together)**	Wilson, 2022 [38]	- CI (Health education)	n/a	n/a	= (vs. CI and BL)	= (vs. CI)	n/a	= (vs. CI)	Parental light physical activity
	Wilson, 2021 [39]	n/a	n/a	n/a	n/a	n/a	n/a	n/a	Family mealtime
	Wilson, 2018 [40]	n/a	n/a	n/a	n/a	n/a	n/a	n/a	Retention in program
**Diabetes Prevention Program among Latino Youths**	Peña, 2022 [50]	- UC (Behavioral modification)	n/a	6-month Weight-related QOL + (vs. BL) and = (vs. UC) 12-month Weight-related QOL + (vs. BL and UC)	= (vs. BL and UC) at 6 months + (vs. BL) and = (vs. UC) at 12 months	n/a	n/a	n/a	Glucose tolerance, insulin sensitivity, insulin secretion, beta-cell function, fat mass, lean mass, HR, SBP, DBP

Legend: + indicates an improvement in the outcome; - indicates a deterioration in the outcome; = indicates the absence of a change in the outcome; vs. BL indicates comparisons were made with intervention baseline measures; vs. CI indicates comparisons were made with a control intervention; vs. WLC indicates comparisons were made with a waitlist control group; vs. UC indicates comparisons were made with the usual care

Abbreviations: *BL* baseline, *CBT *cognitive behavioural therapy, *CI *control intervention, *DBP *diastolic blood pressure, *HR *heart rate, *PA *physical activity, *QOL *quality of life, *SBP *systolic blood pressure, *UC *usual care, *n/a *not applicable (outcome was not measured/reported)

^a ^In the Family Connections study, there were the 3 interventions arms which were informed by FST but were delivered using different formats

As shown in Table 4, among studies that reported on BMI outcomes, virtually all studies with comparisons to baseline values or to waitlist control groups found post-intervention improvements in BMI. For studies that compared BMI to usual care or control interventions, 6 reported improvements, 4 reported no differences, and 1 reported worse outcomes in the FST intervention compared to the control group. For studies examining changes in physical activity, 4 out of 5 studies that used baseline or waitlist control groups reported improvements, whereas only 6 out of 11 studies with usual care or control intervention comparisons reported improvements in physical activity, and other studies reported no differences. For sedentary behaviour outcomes, 3 out of 4 studies using baseline or waitlist controls reported improvements, whereas no differences were found in the 2 studies with usual care or control interventions. Among studies that examined dietary outcomes, most found no difference, except for 2 studies with usual care or control intervention comparisons, and one relying on baseline comparisons. Most studies that reported improvements in mental health outcomes used baseline and waitlist control comparisons, with mixed findings for intervention effects compared to usual care and control interventions. Lastly, of the 10 studies that measured FST concepts (e.g., family communication, family functioning, family support), 5 reported improvements of which 3 were compared to usual care or control interventions, while the other studies reported no differences or mixed findings.

## Discussion

This scoping review sought to describe the use of FST in pediatric obesity management interventions over the past four decades to map current knowledge and identify research gaps and practice implications. Our review reveals that school-aged children and adolescents are more frequently targeted compared to preschoolers and that few interventions specifically target population sub-groups who are at increased risk of obesity and its complications due to systemic barriers to health (e.g., low socioeconomic status, racial/ethnic minority groups). Interventions were most commonly delivered in outpatient hospital settings by multidisciplinary teams using a variety of delivery modalities, and all studies were conducted in Western countries. We identified 11 FST-related concepts that informed intervention components, with parenting skills, family communication, and social/family support being the most common. However, many interventions did not elaborate on how FST was translated into specific intervention components, and few included measurements of FST-related concepts as part of the baseline and post-intervention assessments. Among studies reporting intervention outcomes, BMI was most frequently reported and generally improved following the intervention; however, there were a variety of comparison groups noted ranging from usual care obesity management to psychoeducation and other control interventions. This variety in comparison groups should be considered in the interpretation of intervention effects given differences between studies in intensity and dosage.

Preschool-aged children were infrequently included in the obesity management interventions we reviewed with inconsistent results for BMI, lifestyle behaviours, and/or family systems-related outcomes [51, 53–55, 58, 59]. Considering their young age, it is possible that FST-informed obesity interventions targeting preschool-aged children are more likely to be preventative in nature. Inclusion in this review required children to have overweight/obesity at intervention baseline; exploring the use of FST in the prevention of obesity may shed light on the nature and overall usefulness of FST in preventing obesity among children under 5 years of age.

Moreover, given the higher rates of obesity in some ethnic minority groups [78], culturally adapted FST-informed interventions continue to be a priority. FST concepts integrated in interventions targeting ethnic minority groups did not differ from other interventions, but authors mentioned how cultural considerations and strategies were used to guide implementation. For example, the Supporting Health Interactively through Nutrition (SHINE) study enhanced intervention relevance for African American families through the recruitment of African American providers and community leaders, the usage of photos of African American families in intervention material, and the presentation of data related to African American youth specifically [70]. Other studies used qualitative methods to explore sociocultural values and barriers that could be integrated in the intervention’s final curriculum [35]. Of the 8 interventions that focused on ethnic minorities, 5 included measurements of pre- and post-intervention outcomes (e.g., BMI and lifestyle behaviours), and 4 of these resulted in improvements, lending support to the usefulness of culturally adapted FST-informed interventions.

Almost all studies included in this review reported the involvement of professionals from two or more disciplines. This is in line with the multidisciplinary approach recommended for pediatric obesity management [79]. However, few articles mentioned whether those delivering the interventions were trained in family systems approaches which is essential to ensure appropriate embodiment by involved professionals of core FST intervention components [80, 81]. Interestingly, some interventions included staff outside of the traditional health fields (e.g., parks and recreation staff) which may provide a broader perspective of the different multi-sectoral and multi-systemic factors implicated in pediatric obesity and its solutions [79, 82].

Although most interventions were group-based and were delivered entirely in person, others were either partially or fully delivered virtually using web-based or telephone modalities. Virtual intervention delivery may facilitate reaching more family members, an important consideration from a family systems perspective. Moreover, overall attendance and retention may be improved for interventions delivered virtually [83]. Similarly, the use of home visits was reported in 2 interventions of which one (Multisystemic Therapy) reported effects on outcomes. The latter is one of the few interventions that reported improvements across all measured outcomes, including FST-related concepts, BMI, diet, and adiposity in comparison to a control intervention group [61, 62]. Home visits may be an important modality to consider for the delivery of FST-informed interventions in pediatric obesity management. It has been shown that families support the use of home visits in the context of obesity management and perceive these as having benefits, namely in terms of convenience, tailored care, and family involvement [84]. While previous reviews have highlighted the importance of engaging multiple family members in pediatric obesity management [12], it has been noted that potentially influential family members, such as the other parent (often fathers), siblings, or grandparents, are often neglected in family-based pediatric obesity management interventions [85]. Home-based approaches may facilitate the involvement and engagement of multiple members within a family unit.

BMI outcomes were the most consistently measured to evaluate FST-informed interventions; they also showed the most consistent improvements, notably in comparison to baseline and waitlist control groups but also in comparison to usual care and to non-FST control interventions. These results are in line with previous reviews of family-based interventions that have reported weight-related improvements [10, 14, 86], and lend support to the use of FST-informed interventions in pediatric obesity management. Findings were generally similar with regard to improvements in physical activity but were largely inconsistent for other outcomes. This review highlights the need for more evidence on the benefits of FST-informed interventions in comparison to usual care and standard family-based obesity management interventions not based on FST. There is also a need for evidence on which families and children may benefit the most from FST-informed interventions in comparison to standard obesity management interventions.

Intervention effects on family systems measures (e.g., parenting skills, family communication, etc.) were either not reported or mixed in the few studies that evaluated these outcomes. This is an important knowledge gap given that one of the goals of FST-informed interventions is to improve dynamics and organisation within the family so as to create family environments and conditions that are supportive of improvements in health and lifestyle behaviour changes [11, 12, 87]. Inconsistency in results may be due to the relatively low number of studies that measured FST-related variables. Some studies used qualitative methods to assess participants’ perspectives on changes in the family system following the intervention, both of which reported perceived improvements [36, 57]. Qualitative exploration may allow for a deeper understanding of family beliefs associated with family system concepts at baseline and how these evolve following an intervention. Exploring these perspectives can allow for a more tailored approach to obesity management and can provide a richer understanding of intervention effectiveness related to the family system.

This review highlights the importance of evaluating the family system before and after intervention delivery given its potential role as mediator of intervention effects. Intervening at the family systems level may lead to greater and more sustained changes due to improvements in underlying family dynamics that may hinder or challenge lifestyle modification [12]. In addition, the health of the family system may predict the response to FST-informed obesity management. For example, although Kitzmann et al. did not see improvements in examined family systems concepts following their intervention, baseline parental support for healthy eating habits and positive parenting styles were associated with greater reductions in BMI over the 6-week study [56]. Similarly, Spence et al. found that a healthier family system pre-intervention was associated with improved retention in their program [65].

In order to be included in this review study, studies had to explicitly mention how FST or related theories were used to guide the intervention development. Most studies used FST in combination with other health-related theories to inform certain components of their intervention, but fewer studies used FST as a broader lens through which to approach pediatric obesity at the family system level. Many studies briefly mentioned the use of FST or related theories but lacked a clear embodiment of FST and did not elaborate on the specifics of how these theories were integrated in their intervention delivery. One exception to this was the Families Improving Together (FIT) intervention which was described as deeply rooted in FST [35]. This intervention targeted a number of different FST-related concepts (e.g., parenting skills, family communication) and was centered on creating a positive social climate and promoting warm and supportive family interactions throughout all intervention sessions [35]. It further targeted positive parenting skills through parenting style, parental monitoring, shared decision making, and communication, while promoting family bonding and family support in weekly goal setting [35]. Other interventions that were more explicit on their family systems approaches were the Multisystemic Therapy, which included baseline assessment of the family’s strengths and weaknesses to target individual family needs related to FST concepts [62, 63, 88], the SHINE intervention, which provided detailed and specific descriptions of FST integration in their design [70, 71], and ENTREN-F, which focused on behavioural parenting strategies, parental educational styles, feeding practices, communication skills and adaptive dynamics in the home environment [30].

Previous reviews have also pointed out that existing pediatric obesity interventions based on FST do not fully embody a family systems approach. In their literature review published in 2011, Kitzmann and Beech observed that the majority of pediatric obesity management interventions reviewed had a narrow family focus (e.g., parents were asked to modify health behaviours) while fewer were more broadly family-focused [86]. Additionally, as noted by Skelton et al. in their review of family theories in pediatric obesity management, FST was often used as a theme to discuss pediatric obesity but was rarely used to guide obesity management interventions [12]. Family perspectives and beliefs surrounding the family system were infrequently explored in the studies we reviewed. Exploring these beliefs would allow for a more tailored approach to intervention delivery and would promote an individualized, strengths-based design that builds on a family’s existing values and unique strengths to improve intervention outcomes [89].

Findings from this review provide insight for health care providers seeking to integrate FST into obesity management interventions. FST-informed approaches can be used across the pediatric age groups. Including a combination of in-person and virtual or home-based sessions can facilitate intervening with the family as a whole, and adaptations to increase relevance to specific sociodemographic backgrounds (e.g., socioeconomic status, ethnocultural backgrounds) are key. Training the intervention delivery team in FST and including the assessment of family systems concepts (e.g., baseline and follow-up measures of family communication and family functioning) are essential moving forward.

This review was conducted by a multidisciplinary research team that included health professionals and researchers with expertise in FST and pediatric obesity management as well as a health sciences librarian. We used a broad search strategy to ensure all FST-informed interventions were captured. We included a variety of types of articles such as protocols, intervention descriptions, qualitative studies, randomized controlled trials and quasi-experimental studies. A rigorous approach was used to determine article inclusion/exclusion and to extract data from included studies. For example, a preliminary search guided our final inclusion/exclusion criteria, notably the explicit use of a family systems-related theory in the development of intervention and the focus on obesity management, which allowed us to synthesise evidence from more comparable interventions. In terms of limitations, our review does not include preventive interventions which may have excluded studies targeting preschool-aged children. Additionally, we did not assess the quality of included studies. Although this is not mandatory in scoping reviews, doing so strengthens the synthesised evidence. Lastly, we did not register or publish a protocol for this scoping review.

## Conclusions

This review provides some support for FST as a useful theory to inform the development of pediatric obesity management intervention strategies targeting improvements in obesity-related outcomes, lifestyle behaviours (namely physical activity), and mental health. However, it remains unclear whether improvements at the family system level mediate favourable outcomes. This review further highlights the need for additional evidence on the benefits of FST-informed interventions in comparison to standard family-based obesity management interventions not based on FST. Future research should explore family perspectives and beliefs surrounding FST in pediatric obesity management. Assessing the family system prior to intervening, focusing on the family’s strengths, and exploring beliefs related to the family system may optimize the tailoring of pediatric obesity management interventions to the unique needs and context of each family.

### Supplementary Information

**Supplementary Material 1.** 

**Supplementary Material 2.** 

## Data Availability

The dataset(s) supporting the conclusions of this article are available in the Medline, CINAHL, Embase, and PsycInfo repositories.

## Abbreviations

AD: Adolescent
BL: Baseline
BMI: Body Mass Index
CBT: Cognitive Behavioral Therapy
CI: Control Intervention
CINAHL: Cumulative Index to Nursing and Allied Health Literature
DBP: Diastolic Blood Pressure
DOCS: The Dyadic Communication Scale
FACES IV: The Family Adaptability and Cohesion Evaluation Scales IV
FBBT: Multidisciplinary family-based behavioural therapy for obesity
FERFQ: Family Experiences Related to Food Questionnaire
FIT: Families Improving Together
FIT-T: Families Improving Together-Telehealth
FKFF: Fit Kids / Fit Families
FOTM: Families on the Move
FQ: Family Questionnaire
FST: Family Systems Theory
FUHW: United Families for Health and Wellness
HES-S: Home Environment Survey-Physical Activity
HR: Heart Rate
JBI: Joanna Briggs Institute
LiLi: Lighter Living program
LOOPS: Lund Overweight and Obesity Preschool Study
M + FWL: Motivational + Family Weight Loss Intervention
MEND: Mind, Exercise, Nutrition, Do it!
PA: Physical Activity
PAC: Parents as Agents of Change
PDI-S: The Parenting Dimensions Inventory
PEAS: The Parenting Strategies for Eating and Activity Scale
PRESS: Peer Review of Electronic Search Strategies
PS: Preschool
QOL: Quality of Life
SA: School-Age
SBP: Systolic Blood Pressure
SBT + EP: Standard Behavioural Treatment + Enhanced Parenting
SFI: Self-Report Family Inventory
SHINE: Supporting Health Interactively through Nutrition and Exercise
TAFF: Telephone-based Adiposity prevention for Families
UC: Usual care
UK: United Kingdom
USA: United States of America
WCSS: The Weight Control Strategies Scale
WLC: Wait list control
YQOL: The Youth Quality of Life Inventory

## References

[1] Abarca-Gómez L, Abdeen ZA, Hamid ZA, Abu-Rmeileh NM, Acosta-Cazares B, Acuin C (2017). Worldwide trends in body-mass index, underweight, overweight, and obesity from 1975 to 2016: a pooled analysis of 2416 population-based measurement studies in 128· 9 million children, adolescents, and adults. The Lancet.

[2] Kumar S, Kelly AS (2017). Review of Childhood Obesity: From Epidemiology, Etiology, and Comorbidities to Clinical Assessment and Treatment. Mayo Clin Proc.

[3] Simmonds M, Llewellyn A, Owen CG, Woolacott N (2016). Predicting adult obesity from childhood obesity: a systematic review and meta-analysis. Obes Rev.

[4] Reinehr T (2018). Long-term effects of adolescent obesity: time to act. Nat Rev Endocrinol.

[5] Llewellyn A, Simmonds M, Owen CG, Woolacott N (2016). Childhood obesity as a predictor of morbidity in adulthood: a systematic review and meta-analysis. Obes Rev.

[6] Griffiths LJ, Parsons TJ, Hill AJ (2010). Self-esteem and quality of life in obese children and adolescents: a systematic review. Int J Pediatr Obes.

[7] Epstein LH, Valoski A, Wing RR, McCurley J (1994). Ten-year outcomes of behavioral family-based treatment for childhood obesity. Health psychol.

[8] Epstein LH, Valoski A, Wing RR, McCurley J (1990). Ten-year follow-up of behavioral, family-based treatment for obese children. JAMA.

[9] O’Connor EA, Evans CV, Burda BU, Walsh ES, Eder M, Lozano P (2017). Screening for obesity and intervention for weight management in children and adolescents: evidence report and systematic review for the US Preventive Services Task Force. JAMA.

[10] Sung-Chan P, Sung YW, Zhao X, Brownson RC (2013). Family-based models for childhood-obesity intervention: a systematic review of randomized controlled trials. Obes Rev.

[11] Kaplan SG, Arnold EM, Irby MB, Boles KA, Skelton JA (2014). Family Systems Theory and Obesity Treatment: Applications for Clinicians. Infant Child Adolesc Nutr.

[12] Skelton JA, Buehler C, Irby MB, Grzywacz JG (2012). Where are family theories in family-based obesity treatment?: conceptualizing the study of families in pediatric weight management. Int J Obes (Lond).

[13] Pratt KJ, Skelton JA. Family functioning and childhood obesity treatment: a family systems theory-informed approach. Acad Pediatr. 2018. 10.1016/j.acap.2018.04.001 PMC8111666 29654905

[14] Berge JM, Everts JC (2011). Family-Based Interventions Targeting Childhood Obesity: A Meta-Analysis. Childhood obesity (Print).

[15] Berry D, Sheehan R, Heschel R, Knafl K, Melkus G, Grey M (2004). Family-based interventions for childhood obesity: a review. J Fam Nurs.

[16] Nowicka P, Flodmark CE (2008). Family in pediatric obesity management: a literature review. Int J Pediatr Obes.

[17] Ash T, Agaronov A, Young T, Aftosmes-Tobio A, Davison KK (2017). Family-based childhood obesity prevention interventions: a systematic review and quantitative content analysis. Int J Behav Nutr Phys Act.

[18] Skouteris H, McCabe M, Swinburn B, Newgreen V, Sacher P, Chadwick P (2011). Parental influence and obesity prevention in pre-schoolers: a systematic review of interventions. Obes Rev.

[19] Joanna Briggs Institute. Joanna Briggs Institute Australia: The University of Adelaide; 2018. Available from: http://joannabriggs.org/.

[20] Tricco AC, Lillie E, Zarin W, O'Brien KK, Colquhoun H, Levac D (2018). PRISMA Extension for Scoping Reviews (PRISMA-ScR): Checklist and Explanation. Ann Intern Med.

[21] Rethlefsen ML, Kirtley S, Waffenschmidt S, Ayala AP, Moher D, Page MJ (2021). PRISMA-S: an extension to the PRISMA Statement for Reporting Literature Searches in Systematic Reviews. Syst Rev.

[22] McGowan J, Sampson M, Salzwedel DM, Cogo E, Foerster V, Lefebvre C (2016). PRESS Peer Review of Electronic Search Strategies: 2015 Guideline Statement. J Clin Epidemiol.

[23] Bramer WM, Giustini D, de Jonge GB, Holland L, Bekhuis T (2016). De-duplication of database search results for systematic reviews in EndNote. J Med Libr Assoc.

[24] Ouzzani M, Hammady H, Fedorowicz Z, Elmagarmid A (2016). Rayyan-a web and mobile app for systematic reviews. Syst Rev.

[25] Doherty WJ, Harkaway JE (1990). Obesity and Family Systems: A family FIRO approach to assessment and treatment planning. J Marital Fam Ther.

[26] Ouzzani M, Hammady H, Fedorowicz Z, Elmagarmid A (2016). Rayyan—a web and mobile app for systematic reviews. Syst Rev.

[27] Bengtsson M (2016). How to plan and perform a qualitative study using content analysis. NursingPlus Open.

[28] Appelhans BM, French SA, Bradley LE, Lui K, Janssen I, Richardson D (2020). CHECK: A randomized trial evaluating the efficacy and cost-effectiveness of home visitation in pediatric weight loss treatment. Contemp Clin Trials.

[29] Dilley JR, Singletary CR, Ard JD, Giles S, Skelton JA, Heboyan V (2020). Protocol for a randomized controlled feasibility study of a coordinated parent/child weight loss intervention: Dyad Plus. Transl J Am Coll Sports Med.

[30] Rojo M, Lacruz T, Solano S, Vivar M, Del Río A, Martínez J (2022). ENTREN-F family-system based intervention for managing childhood obesity: Study protocol for a randomized controlled trial at primary care. Obes Res Clin Pract.

[31] Rojo M, Lacruz T, Solano S, Gutiérrez A, Beltrán-Garrayo L, Veiga OL (2022). Family-reported barriers and predictors of short-term attendance in a multidisciplinary intervention for managing childhood obesity: A psycho-family-system based randomised controlled trial (ENTREN-F). Eur Eat Disord Rev.

[32] Christison AL, Evans TA, Bleess BB, Wang H, Aldag JC, Binns HJ (2016). Exergaming for health: A randomized study of community-based exergaming curriculum in pediatric weight management. Games Health J.

[33] Prado G, Fernandez A, St George SM, Lee TK, Lebron C, Tapia MI (2020). Results of a Family-Based Intervention Promoting Healthy Weight Strategies in Overweight Hispanic Adolescents and Parents: An RCT. Am J Prev Med.

[34] Perrino T, Brincks AM, Estrada Y, Messiah SE, Prado G (2022). Reducing Screen-Based Sedentary Behavior Among Overweight and Obese Hispanic Adolescents Through a Family-Based Intervention. J Phys Act Health.

[35] Alia KA, Wilson DK, McDaniel T, George SMS, Kitzman-Ulrich H, Smith K (2015). Development of an innovative process evaluation approach for the Families Improving Together (FIT) for weight loss trial in African American adolescents. Eval Program Plann.

[36] Sweeney AM, Wilson DK, Loncar H, Brown A (2019). Secondary benefits of the families improving together (FIT) for weight loss trial on cognitive and social factors in African American adolescents. Int J Behav Nutr Phys Act.

[37] Wilson DK, Kitzman-Ulrich H, Resnicow K, Van Horn ML, George SMS, Siceloff ER (2015). An overview of the Families Improving Together (FIT) for weight loss randomized controlled trial in African American families. Contemp Clin Trials.

[38] Wilson DK, Sweeney AM, Van Horn ML, Kitzman H, Law LH, Loncar H (2022). The Results of the Families Improving Together (FIT) for Weight Loss Randomized Trial in Overweight African American Adolescents. Annals of behavioral medicine : a publication of the Society of Behavioral Medicine.

[39] Wilson DK, Sweeney AM, Quattlebaum M, Loncar H, Kipp C, Brown A (2021). The Moderating Effects of the Families Improving Together (FIT) for Weight Loss Intervention and Parenting Factors on Family Mealtime in Overweight and Obese African American Adolescents. Nutrients.

[40] Wilson DK, Sweeney AM, Law LH, Kitzman-Ulrich H, Resnicow K (2018). Web-Based Program Exposure and Retention in the Families Improving Together for Weight Loss Trial. Ann Behav Med.

[41] Quattlebaum M, Wilson DK, Sweeney AM, Zarrett N (2021). Moderating Effects of Parental Feeding Practices and Emotional Eating on Dietary Intake among Overweight African American Adolescents. Nutrients.

[42] Biggs BK, Rodgers KV, Nayman SJ, Hofschulte DR, Loncar H, Kumar S (2023). Translation of a family-based behavioral intervention for adolescent obesity using the RE-AIM framework and common steps from adaptation frameworks. Translational behavioral medicine.

[43] James KS, Connelly CD, Rutkowski E, McPherson D, Gracia L, Mareno N (2008). Family-based weight management with latino mothers and children. J Spec Pediatr Nurs.

[44] Estabrooks PA, Shoup JA, Gattshall M, Dandamudi P, Shetterly S, Xu S (2009). Automated telephone counseling for parents of overweight children: a randomized controlled trial. Am J Prev Med.

[45] Zoellner JM, You W, Hill JL, Brock DJP, Yuhas M, Alexander RC (2019). A comparative effectiveness trial of two family-based childhood obesity treatment programs in a medically underserved region: Rationale, design & methods. Contemp Clin Trials.

[46] Zoellner JM, You W, Hill JL, Brock DJP, Yuhas M, Price B (2022). Comparing two different family-based childhood obesity treatment programmes in a medically underserved region: Effectiveness, engagement and implementation outcomes from a randomized controlled trial. Pediatric obesity.

[47] Nowicka P, Höglund P, Pietrobelli A, Lissau I, Flodmark C-E (2008). Family Weight School treatment: 1-year results in obese adolescents. Int J Pediatr Obes.

[48] Joosse L, Stearns M, Anderson H, Hartlaub P, Euclide J (2008). Fit Kids/Fit Families: a report on a countywide effort to promote healthy behaviors. Wis Med J.

[49] Soltero EG, Konopken YP, Olson ML, Keller CS, Castro FG, Williams AN (2017). Preventing diabetes in obese Latino youth with prediabetes: a study protocol for a randomized controlled trial. BMC Public Health.

[50] Peña A, Olson ML, Hooker E, Ayers SL, Castro FG, Patrick DL (2022). Effects of a Diabetes Prevention Program on Type 2 Diabetes Risk Factors and Quality of Life Among Latino Youths With Prediabetes: A Randomized Clinical Trial. JAMA Netw Open.

[51] Orban K, Edberg A-K, Thorngren-Jerneck K, Önnerfält J, Erlandsson L-K (2014). Changes in parents’ time use and its relationship to child obesity. Phys Occup Ther Pediatr.

[52] Önnerfält J, Erlandsson L-K, Orban K, Broberg M, Helgason C, Thorngren-Jerneck K (2012). A family-based intervention targeting parents of preschool children with overweight and obesity: conceptual framework and study design of LOOPS-Lund overweight and obesity preschool study. BMC Public Health.

[53] Wilson TA, Liu Y, Adolph AL, Sacher PM, Barlow SE, Pont S (2019). Behavior modification of diet and parent feeding practices in a community-Vs primary care–centered intervention for childhood obesity. JNEB..

[54] Law C, Cole T, Cummins S, Fagg J, Morris S, Roberts H. A pragmatic evaluation of a family-based intervention for childhood overweight and obesity. Public Health Res. 2014;2(5). 27466647

[55] Sacher PM, Kolotourou M, Chadwick PM, Cole TJ, Lawson MS, Lucas A (2010). Randomized controlled trial of the MEND program: a family-based community intervention for childhood obesity. Obesity.

[56] Kitzman-Ulrich H, Wilson DK, St. George SM, Segal M, Schneider E, Kugler K (2011). A preliminary test of a motivational and parenting weight loss program targeting low-income and minority adolescents. Child Obes..

[57] Chamay Weber C, Camparini N, Lanza L, Narring F (2016). Parents’ integration in the treatment of adolescents with obesity: A qualitative study. Fam Syst Health.

[58] Bocca G, Kuitert M, Sauer P, Stolk R, Flapper B, Corpeleijn E (2014). A multidisciplinary intervention programme has positive effects on quality of life in overweight and obese preschool children. Acta Paediatr.

[59] Bocca G, Kuitert MW, Sauer PJ, Corpeleijn E (2018). Effect of a multidisciplinary treatment program on eating behavior in overweight and obese preschool children. J Pediatr Endocrinol Metab.

[60] Kitzman-Ulrich H, Hampson R, Wilson DK, Presnell K, Brown A, O'Boyle M (2009). An adolescent weight-loss program integrating family variables reduces energy intake. J Am Diet Assoc.

[61] Ellis DA, Janisse H, Naar-King S, Kolmodin K, Jen KLC, Cunningham P (2010). The effects of multisystemic therapy on family support for weight loss among obese African-American adolescents: findings from a randomized controlled trial. J Dev Behav Pediatr..

[62] Naar-King S, Ellis D, Kolmodin K, Cunningham P, Jen KLC, Saelens B (2009). A randomized pilot study of multisystemic therapy targeting obesity in African-American adolescents. J Adolesc Health..

[63] Idalski Carcone A, MacDonell KE, Naar-King S, Ellis DA, Cunningham PB, Kaljee L (2011). Treatment engagement in a weight loss intervention for African American adolescents and their families. Child Health Care.

[64] Flodmark C-E, Ohlsson T, Rydén O, Sveger T (1993). Prevention of progression to severe obesity in a group of obese schoolchildren treated with family therapy. Pediatrics.

[65] Spence ND, Newton AS, Keaschuk RA, Ambler KA, Jetha MM, Holt NL (2017). Predictors of short-and long-term attrition from the parents as agents of change randomized controlled trial for managing pediatric obesity. J Pediatr Health Care.

[66] Ball GD, Mushquash AR, Keaschuk RA, Ambler KA, Newton AS (2017). Using Intervention Mapping to develop the Parents as Agents of Change (PAC©) intervention for managing pediatric obesity. BMC Res Notes.

[67] Ball GD, Ambler KA, Keaschuk RA, Rosychuk RJ, Holt NL, Spence JC (2012). Parents as Agents of Change (PAC) in pediatric weight management: The protocol for the PAC randomized clinical trial. BMC Pediatr.

[68] Spence ND, Newton AS, Keaschuk RA, Ambler KA, Holt NL, Jetha MM (2023). Parents as Agents of Change in Managing Pediatric Obesity: A Randomized Controlled Trial Comparing Cognitive Behavioral Therapy versus Psychoeducation Interventions. Childhood obesity (Print).

[69] Steele RG, Aylward BS, Jensen CD, Cushing CC, Davis AM, Bovaird JA (2011). Comparison of a family-based group intervention for youths with obesity to a brief individual family intervention: A practical clinical trial of positively fit. J Pediatr Psychol.

[70] St. George SM, Wilson DK, Van Horn ML (2018). Project SHINE: Effects of a randomized family-based health promotion program on the physical activity of African American parents. J Behav Med..

[71] St. George SM, Wilson DK, Schneider EM, Alia KA (2013). Project SHINE: Effects of parent–adolescent communication on sedentary behavior in African American adolescents. J Pediatr Psychol.

[72] Nowicka P, Pietrobelli A, Flodmark C-E (2007). Low-intensity family therapy intervention is useful in a clinical setting to treat obese and extremely obese children. Int J Pediatr Obes.

[73] Hadley W, McCullough MB, Rancourt D, Barker D, Jelalian E (2015). Shaking up the system: the role of change in maternal-adolescent communication quality and adolescent weight loss. J Pediatr Psychol.

[74] Jelalian E, Hadley W, Sato A, Kuhl E, Rancourt D, Oster D (2015). Adolescent weight control: An intervention targeting parent communication and modeling compared with minimal parental involvement. J Pediatr Psychol.

[75] Herget S, Markert J, Petroff D, Gausche R, Grimm A, Hilbert A (2015). Psychosocial well-being of adolescents before and after a 1-year telephone-based adiposity prevention study for families. J Adolesc Health.

[76] Markert J, Herget S, Petroff D, Gausche R, Grimm A, Kiess W (2014). Telephone-based adiposity prevention for families with overweight children (TAFF-Study): one year outcome of a randomized, controlled trial. IJERPH.

[77] Markert J, Alff F, Zschaler S, Gausche R, Kiess W, Blüher S (2013). Prevention of childhood obesity: Recruiting strategies via local paediatricians and study protocol for a telephone-based counselling programme. Obes Res Clin Pract.

[78] Ogden CL, Carroll MD, Kit BK, Flegal KM (2012). Prevalence of obesity and trends in body mass index among US children and adolescents, 1999–2010. JAMA.

[79] Lau DC, Douketis JD, Morrison KM, Hramiak IM, Sharma AM, Ur E (2007). 2006 Canadian clinical practice guidelines on the management and prevention of obesity in adults and children [summary]. CMAJ.

[80] Sveinbjarnardottir EK, Svavarsdottir EK, Saveman BI (2011). Nurses attitudes towards the importance of families in psychiatric care following an educational and training intervention program. J Psychiatr Ment Health Nurs.

[81] Larivaara P, Väisänen E, Väisänen L, Kiuttu J (1995). Training general practitioners in family systems medicine. Nord J Psychiatry.

[82] Bischoff SC, Boirie Y, Cederholm T, Chourdakis M, Cuerda C, Delzenne NM (2017). Towards a multidisciplinary approach to understand and manage obesity and related diseases. Clinical nutr.

[83] Moorman EL, Koskela-Staples NC, Mathai BB, Fedele DA, Janicke DM. Pediatric obesity treatment via telehealth: current evidence and future directions. Curr Obes Rep. 2021:1–14. 10.1007/s13679-021-00446-w 34302603

[84] Gehring ND, Ball GD, Perez A, Holt NL, Neuman D, Spence N (2018). Families’ perceived benefits of home visits for managing paediatric obesity outweigh the potential costs and barriers. Acta Paediatr.

[85] May C, Chai LK, Burrows T (2017). Parent, partner, co-parent or partnership? The need for clarity as family systems thinking takes hold in the quest to motivate behavioural change. Children.

[86] Kitzmann KM, Beech BM (2011). Family-based interventions for pediatric obesity: methodological and conceptual challenges from family psychology. Couple Family Psychol.

[87] Kitzman-Ulrich H, Wilson DK, George SMS, Lawman H, Segal M, Fairchild A (2010). The integration of a family systems approach for understanding youth obesity, physical activity, and dietary programs. Clin Child Fam Psychol Rev.

[88] Ellis DA, Janisse H, Naar-King S, Kolmodin K, Jen KL, Cunningham P (2010). The effects of multisystemic therapy on family support for weight loss among obese African-American adolescents: findings from a randomized controlled trial. Journal of developmental and behavioral pediatrics : JDBP.

[89] Rhee KE, Lumeng JC, Appugliese DP, Kaciroti N, Bradley RH (2006). Parenting styles and overweight status in first grade. Pediatrics.

