# Supplementary material for: Family systems approaches in pediatric obesity management: a scoping review
Source: BMC Pediatr. 2024 Apr 2;24:235. doi: 10.1186/s12887-024-04646-w (PMC10985863; doi:10.1186/s12887-024-04646-w)
Supplement: Supplementary file 1 — Supplementary Material 1. [file 12887_2024_4646_MOESM1_ESM.pdf]

Title: Family systems approaches in pediatric obesity management: A scoping review
Authors: N Wills-Ibarra, K Chemtob, H Hart, F Frati, KJ Pratt, GD Ball, A Van Hulst

Supplemental File 1. Detailed search strategy implemented in MEDLINE, CINAHL, EMBASE and PSYCHINFO

A) MEDLINE search conducted on April 2 2020 and updated on October 27, 2023

1. Family Health/
2. exp Family relations/
3. Parenting/
4. exp Parent-Child Relations/
5. Family Therapy/
6. Family Systems Theory.tw,kf.
7. Circumplex Model of Family Functioning.tw,kf.
8. Double ABCX Model of Family Stress.tw,kf.
9. Family Stress Model of Economic Strain.tw,kf.
10. Family Development Theory.tw,kf.
11. Ecologic Systems Theory.tw,kf.
12. General Systems Theory.tw,kf.
13. Calgary Family Assessment Intervention Model.tw,kf.
14. ("Calgary Family" adj 2 model).tw,kf.
15. Structural Family Therapy.tw,kf.
16. Emotion-Focused Family Model.tw,kf.
17. (family adj3 (based or function* or communicat* or cohesion or system* or model* or approach* or dynamic* or cohesion or organiz* or theory or theories)).tw,kf.
18. 1 or 2 or 3 or 4 or 5 or 6 or 7 or 8 or 9 or 10 or 11 or 12 or 13 or 14 or 15 or 16 or 17
19. Adolescent/
20. exp Child/
21. 19 or 20
22. Obesity/ or Overweight/
23. 21 and 22
24. Pediatric Obesity/
25. ((obesity or obese or overweight or adiposity) adj10 (child* or p?ediatric or youth or adolescent* or teen* or preteen* or pre-teen* or toddler* or preschooler* or juvenile)).tw,kf,jw.
26. 23 or 24 or 25
27. 18 and 26

B) CINAHL search conducted on April 2 2020

#	Query	Results
S33	S31 AND S32	2,440
S32	EM 1930-20200402	7,185,153

S31	S6 AND S30	2,531
S30	S7 OR S8 OR S9 OR S10 OR S11 OR S12 OR S13 OR S14 OR S15 OR S16 OR S17 OR S18 OR S19 OR S20 OR S21 OR S22 OR S23 OR S24 OR S25 OR S26 OR S27 OR S28 OR S29	113,279
S29	TI (family N3 (based or function* or communicat* or cohesion or system* or model* or approach* or dynamic* or cohesion or organiz* or theory or theories))) OR AB (family N3 (based or function* or communicat* or cohesion or system* or model* or approach* or dynamic* or cohesion or organiz* or theory or theories)))	26,513
S28	TI Family Stress Model of Economic Strain OR AB Family Stress Model of Economic Strain	Display
S27	TI Family Stress Model of Economic Strain OR AB Family Stress Model of Economic Strain	Display
S26	TI Double ABCX Model of Family Stress OR AB Double ABCX Model of Family Stress	Display
S25	TI Circumplex Model of Family Functioning OR AB Circumplex Model of Family Functioning	2
S24	TI Family Systems Theory OR AB Family Systems Theory	Display
S23	(MH "Parent-Child Relations+")	Display
S22	(MH "Family Therapy") OR (MH "Family Therapy (Iowa NIC)")	Display
S21	TI Emotion-Focused Family Model OR AB Emotion-Focused Family Model	Display
S20	TI Emotion-Focused Family Model OR AB Emotion-Focused Family Model	Display
S19	TI Emotion-Focused Family Therapy OR AB Emotion-Focused Family Therapy	Display
S18	TI Structural Family Therapy OR AB Structural Family Therapy	Display
S17	TI "Calgary Family" N2 model OR AB "Calgary Family" N2 model	Display
S16	TI Calgary Family Assessment Intervention Model OR AB Calgary Family Assessment Intervention Model	Display
S15	TI Calgary Family Assessment Intervention Model OR AB Calgary Family Assessment Intervention Model	Display

S14	TI General Systems Theory OR AB General Systems Theory	Display
S13	TI Ecologic Systems Theory OR AB Ecologic Systems Theory	Display
S12	(MH "Child Rearing")	Display
S11	TI Ecologic Systems Theory OR AB Ecologic Systems Theory	Display
S10	TI Family Development Theory OR AB Family Development Theory	Display
S9	(MH "Parenting")	Display
S8	(MH "Family Health")	Display
S7	(MH "Family Functioning+") OR (MH "Family Relations+")	Display
S6	S3 OR S4 OR S5	40,420
S5	TI ((obesity or obese or overweight or adiposity) N10 (child* or p?ediatric or youth or adolescent* or teen* or preteen* or pre-teen* or toddler* or preschooler* or juvenile)) OR AB ((obesity or obese or overweight or adiposity) N10 (child* or p?ediatric or youth or adolescent* or teen* or preteen* or pre-teen* or toddler* or preschooler* or juvenile))	Display
S4	(MH "Pediatric Obesity")	Display
S3	S1 AND S2	20,666
S2	(MH "Obesity") OR (MH "Obesity, Morbid")	Display
S1	(MH "Adolescence+") OR (MH "Child") OR (MH "Child, Abandoned") OR (MH "Child, Disabled") OR (MH "Child, Gifted") OR (MH "Child, Institutionalized") OR (MH "Child, Medically Fragile") OR (MH "Child, Preschool") OR (MH "Latchkey Children") OR (MH "Only Child")	Display

C) EMBASE search conducted on June 2 2020

1. parenthood/ or child rearing/
2. family relation/ or exp child parent relation/ or family conflict/
3. family counseling/ or parent counseling/
4. family health/
5. (family adj3 (based or function* or communicat* or cohesion or system* or model* or approach* or dynamic* or cohesion or organiz* or theory or theories)).tw,kw.
6. Family Systems Theory.tw,kw.

7. Circumplex Model of Family Functioning.tw,kw.
8. Double ABCX Model of Family Stress.tw,kw.
9. Family Stress Model of Economic Strain.tw,kw.
10. Family Development Theory.tw,kw.
11. Ecologic Systems Theory.tw,kw.
12. General Systems Theory.tw,kw.
13. Calgary Family Assessment Intervention Model.tw,kw.
14. Structural Family Therapy.tw,kw.
15. ("Calgary Family" adj 2 model).tw,kw.
16. Emotion-Focused Family Model.tw,kw.
17. 1 or 2 or 3 or 4 or 5 or 6 or 7 or 8 or 9 or 10 or 11 or 12 or 13 or 14 or 15 or 16
18. adolescent/
19. exp child/
20. 18 or 19
21. obesity/
22. 20 and 21
23. childhood obesity/
24. ((obesity or obese or overweight or adiposity) adj10 (child* or p?ediatric or youth or adolescent* or teen* or preteen* or pre-teen* or toddler* or preschooler* or juvenile)).tw,kw.
25. 22 or 23 or 24
26. 17 and 25

D) PSYCHINFO search conducted on June 2 2020

1. exp parenting/ or childrearing attitudes/ or parental attitudes/ or parental characteristics/
2. Family Systems Theory/
3. exp family therapy/
4. Family Intervention/
5. (family adj3 (based or function* or communicat* or cohesion or system* or model* or approach* or dynamic* or cohesion or organiz* or theory or theories)).tw.
6. Family Systems Theory.tw.
7. Circumplex Model of Family Functioning.tw.
8. Double ABCX Model of Family Stress.tw.
9. Family Stress Model of Economic Strain.tw.
10. Family Development Theory.tw.
11. Ecologic Systems Theory.tw.
12. General Systems Theory.tw.
13. Calgary Family Assessment Intervention Model.tw.
14. Structural Family Therapy.tw.
15. ("Calgary Family" adj 2 model).tw.

16. Emotion-Focused Family Model.tw.
17. 1 or 2 or 3 or 4 or 5 or 6 or 7 or 8 or 9 or 10 or 11 or 12 or 13 or 14 or 15 or 16
18. exp adolescent development/ or adolescent health/
19. exp childhood development/
20. 18 or 19
21. obesity/
22. 20 and 21
23. ((obesity or obese or overweight or adiposity) adj10 (child* or p?ediatric or youth or adolescent* or teen* or preteen* or pre-teen* or toddler* or preschooler* or juvenile)).tw.
24. 22 or 23
25. 17 and 24
26. limit 25 to (english or french or spanish)
27. limit 26 to yr="1980 -Current"
