# Supplementary material for: Family systems approaches in pediatric obesity management: a scoping review
Source: BMC Pediatr. 2024 Apr 2;24:235. doi: 10.1186/s12887-024-04646-w (PMC10985863; doi:10.1186/s12887-024-04646-w)
Supplement: Supplementary file 2 — Supplementary Material 2. [file 12887_2024_4646_MOESM2_ESM.pdf]

Title: Family systems approaches in pediatric obesity management: A scoping review **Authors:** N Wills-Ibarra, K Chemtob, H Hart, F Frati, KJ Pratt, GD Ball, A Van Hulst

Supplemental Table 2: Description of n=50 obesity management intervention studies included by intervention name

Intervention/ program name	Author and year	Description vs. Evaluation	Total sample size at baseline	Study design	FST/model that guided intervention development	Other theories that guided intervention development
**Creating Health Environments for Chicago Kids (CHECK) Trial**	Appelhans, 2020[1]	Description	266 children and their parents/guardians	RCT	Socioecological Model Parents as Agents of Change	n/a
**Dyad plus**	Dilley, 2020[2]	Description	45 parent-adolescent dyads	Pilot RCT	FST, Familial Approach to the Treatment of Childhood Obesity	Social Cognitive Theory, Self-Determination Theory
**ENTREN-F**	Rojo, 2022[3]	Description	165 children and their parents/guardians	RCT protocol	FST	Cognitive Behavioural Therapy
	Rojo, 2022[4]	Evaluation	165 children and their parents/guardians	Secondary analysis of a RCT	FST	Cognitive Behavioural Therapy
**Exergaming for Health**	Christison, 2016[5]	Evaluation	80 children and their parents/guardians	RCT	FST	Social Cognitive Theory
**Familias Unidas (United Families for Health and Wellness-FUHW)**	Prado, 2020[6]	Evaluation	280 adolescents and their parents	RCT	FST, Eco-developmental framework	n/a
	Perrino, 2022[7]	Evaluation	280 adolescents and their parents	Secondary analysis of a RCT	FST, Eco-developmental framework	n/a
**Families Improving Together (FIT)**	Alia, 2015[8]	Description	19 adolescent-parent dyads	Process evaluation of RCT	FST	Self-Determination Theory, Social Cognitive Theory
	Sweeney, 2019[9]	Evaluation	41 adolescents and 41 parents	Qualitative study	FST	Self-Determination Theory, Social Cognitive Theory
	Wilson, 2015[10]	Description	520 families (expected)	RCT Protocol	FST	Self-Determination Theory, Social Cognitive Theory
	Wilson, 2018[11]	Evaluation	125 parent-adolescent dyads	RCT	FST	Self-Determination Theory, Social Cognitive Theory
	Wilson, 2021[12]	Evaluation	241 parent/caregiver-adolescent dyads	RCT	FST	Self-Determination Theory, Social Cognitive Theory
	Quattlebaum, 2021[13]	Evaluation	127 parent-adolescent dyads	Secondary analysis of baseline data of a RCT	FST	n/a
	Wilson, 2022[14]	Evaluation	241 adolescent-caregiver dyads	RCT	FST	Self-Determination Theory, Social Cognitive Theory
**Families Improving Together-**	Biggs, 2023[15]	Description	6 families	Pilot RCT	FST	Social Cognitive Theory, Self-Determination Theory

**Telehealth (FIT-T)**						
**Families on the Move (FOTM)**	James, 2008[16]	Description	18 children and 14 mothers	Pilot feasibility study using mixed methods	FST	The Family Based Health Centered Approach (Golan & Weizman, 2001)
**Family Connections**	Estabrooks, 2009[17]	Evaluation	220 parent-child dyads	RCT	Social Ecologic Theory (model proposed by Golan et al. - Parents as Agents of Change)	n/a
	Zoellner, 2019[18]	Description	116-150 (expected)	RCT Protocol	Social Ecologic Theory (model proposed by Golan et al. - Parents as Agents of Change)	n/a
	Zoellner, 2022[19]	Evaluation	139 children	RCT	Social Ecologic Theory (model proposed by Golan et al. - Parents as Agents of Change)	n/a
**Family Weight School Model**	Nowicka, 2008[20]	Evaluation	72 adolescents and their parents	Quasi-experimental design (with control group, no randomization)	Systemic Family Therapy	Solution-focused Therapy (Shazer, Berg & colleagues)
**Fit Kids / Fit Families (FKFF)**	Joosse, 2008[21]	Evaluation	68 children and their families	Pre- and post-analysis of intervention	FST	n/a
**Diabetes Prevention Program among Latino Youths**	Soltero, 2017[22]	Description	120 adolescents (expected)	RCT Protocol	Expanded Eco-developmental Model	Social Cognitive Theory
	Peña, 2022[23]	Evaluation	117 adolescents and their parents	RCT	Expanded Eco-developmental Model	Social Cognitive Theory
**Lighter Living program (LiLi)**	Orban, 2014[24]	Evaluation	30 parents and 17 children	RCT	Systems Theory	ValMO model and an eco-cultural perspective
**Lund Overweight and Obesity Preschool Study (LOOPS)**	Onnerfalt, 2012[25]	Description	240 parents (expected)	RCT protocol	FST / Ecological systems theory	N/A
	Law, 2014[26]	Evaluation	13,998 children	Mixed methods	Systems Theory	Learning Theory, Social Cognitive Theory

**Mind, Exercise, Nutrition, Do it! (MEND)**	Sacher, 2010[27]	Evaluation	116 children and their parents	RCT	Systems Theory	Learning Theory, Social Cognitive Theory
	Wilson, 2019[28]	Evaluation	549 children and their families	Secondary analysis of RCT	Social Ecological Model	Social Cognitive Theory
**Motivational + family weight loss intervention (M + FWL)**	Kitzman-Ulrich, 2011[29]	Evaluation	20 families	Pilot RCT	FST	Self-Determination Theory, Cognitive Dissonance Theory
**Multidisciplinary family-based behavioural therapy for obesity (FBBT)**	Chamay Weber, 2016[30]	Evaluation	23 parents and 21 adolescents	Qualitative study	FST	Cognitive Behavioural Theory
**Multidisciplinary Treatment Program**	Bocca, 2014[31]	Evaluation	75 children	RCT	FST	Developmental Psychology, Learning Theory
	Bocca, 2018[32]	Evaluation	75 children	RCT	FST	Developmental Psychology, Learning Theory
**Multifamily Therapy plus Pyschoeducation**	Kitzman-Ulrich, 2009[33]	Evaluation	42 adolescent and their parents	RCT	FST	Social Cognitive Theory
**Multisystemic Therapy**	Ellis, 2010[34]	Evaluation	49 adolescents and their families	Pilot RCT with repeated measures design	Multisystemic Therapy	Cognitive Behavioural Theory
	Idalski Carcone, 2011[35]	Description	10 adolescents and their parents	Qualitative phenomenology	Multisystemic Therapy	n/a
	Naar-King, 2009[36]	Evaluation	49 adolescents and their caregivers	Pilot RCT	Multisystemic Therapy, Social-ecological Theory	Cognitive Behavioural Theory
**No name**	Flodmark, 1993[37]	Evaluation	94 children	Clinical Trial	FST	n/a
**Parents as Agents of Change (PAC)**	Ball, 2017[38]	Description	58 families	Intervention mapping & Pilot study	FST	Cognitive Behavioural Theory
	Ball, 2012[39]	Description	90 families (expected)	RCT Protocol	FST / Parents as Agents of Change	n/a

	Spence, 2017[40]	Evaluation	52 families	RCT	FST/Parents as Agents of Change; Ecologic Systems Theory	Cognitive Behavioural Theory
	Spence, 2023[41]	Evaluation	52 families	RCT	FST/Parents as Agents of Change; Ecologic Systems Theory	Cognitive Behavioural Theory
**Positively Fit**	Steele, 2011[42]	Evaluation	93 families	RCT	Multisystemic Theory	n/a
**SHINE**	St George, 2013[43]	Evaluation	73 adolescents-caregiver dyads	Multiple Cohort RCT	FST	Social Cognitive Theory, Self-Determination Theory
	St George, 2018[44]	Evaluation	89 adolescents and their parents	Multiple Cohort RCT	FST	Social Cognitive Theory, Self-Determination Theory
**Solution-focused family therapy**	Nowicka, 2007[45]	Evaluation	44 children and their parents	Pre- and post-analysis of intervention	Systemic Family Therapy	Solution-focused Therapy (Shazer, Berg & colleagues)
**Standard Behavioral Treatment + Enhanced Parenting (SBT + EP)**	Hadley, 2015[46]	Evaluation	38 mother-adolescent dyads	Qualitative study post RCT	FST	n/a
	Jelalian, 2015[47]	Evaluation	49 adolescents and a caregiver	RCT	FST	n/a
**T.A.F.F. (Telephone based Adiposity prevention For Families) study**	Herget, 2015[48]	Evaluation	154 adolescents	RCT	Solution-Focused Systemic Therapy	n/a
	Markert, 2014[49]	Evaluation	303 children and their families	RCT	Solution-Focused Systemic Therapy	n/a
	Markert, 2013[50]	Description	303 candidates started the intervention	RCT Protocol	Systems Theory	n/a

References

1. Appelhans BM, French SA, Bradley LE, et al. CHECK: A randomized trial evaluating the efficacy and cost-effectiveness of home visitation in pediatric weight loss treatment. *Contemp Clin Trials* 2020;88:105891.

2. Dilley JR, Singletary CR, Ard JD, et al. Protocol for a randomized controlled feasibility study of a coordinated parent/child weight loss intervention: Dyad Plus. *Transl J Am Coll Sports Med* 2020;5(12).

3. Rojo M, Lacruz T, Solano S, et al. ENTREN-F family-system based intervention for managing childhood obesity: Study protocol for a randomized controlled trial at primary care. *Obesity research & clinical practice* 2022;16(4):319-329.

4. Rojo M, Lacruz T, Solano S, et al. Family-reported barriers and predictors of short-term attendance in a multidisciplinary intervention for managing childhood obesity: A psycho-family-system based randomised controlled trial (ENTREN-F). *Eur Eat Disord Rev* 2022;30(6):746-759.

5. Christison AL, Evans TA, Bleess BB, et al. Exergaming for health: A randomized study of community-based exergaming curriculum in pediatric weight management. *Games Health J* 2016;5(6):413-421.

6. Prado G, Fernandez A, St George SM, et al. Results of a Family-Based Intervention Promoting Healthy Weight Strategies in Overweight Hispanic Adolescents and Parents: An RCT. *Am J Prev Med* 2020;59(5):658-668.
7. Perrino T, Brincks AM, Estrada Y, et al. Reducing Screen-Based Sedentary Behavior Among Overweight and Obese Hispanic Adolescents Through a Family-Based Intervention. *J Phys Act Health* 2022;19(7):509-517.
8. Alia KA, Wilson DK, McDaniel T, et al. Development of an innovative process evaluation approach for the Families Improving Together (FIT) for weight loss trial in African American adolescents. *Eval. Program Plann.* 2015;49:106-116.
9. Sweeney AM, Wilson DK, Loncar H, et al. Secondary benefits of the families improving together (FIT) for weight loss trial on cognitive and social factors in African American adolescents. *Int J Behav Nutr Phys Act* 2019;16(1):1-10.
10. Wilson DK, Kitzman-Ulrich H, Resnicow K, et al. An overview of the Families Improving Together (FIT) for weight loss randomized controlled trial in African American families. *Contemp. Clin. Trials* 2015;42:145-157.
11. Wilson DK, Sweeney AM, Law LH, et al. Web-Based Program Exposure and Retention in the Families Improving Together for Weight Loss Trial. *Annals of Behavioral Medicine* 2018;53(4):399-404.
12. Wilson DK, Sweeney AM, Quattlebaum M, et al. The Moderating Effects of the Families Improving Together (FIT) for Weight Loss Intervention and Parenting Factors on Family Mealtime in Overweight and Obese African American Adolescents. *Nutrients* 2021;13(6).
13. Quattlebaum M, Wilson DK, Sweeney AM, et al. Moderating Effects of Parental Feeding Practices and Emotional Eating on Dietary Intake among Overweight African American Adolescents. *Nutrients* 2021;13(6).
14. Wilson DK, Sweeney AM, Van Horn ML, et al. The Results of the Families Improving Together (FIT) for Weight Loss Randomized Trial in Overweight African American Adolescents. *Annals of behavioral medicine : a publication of the Society of Behavioral Medicine* 2022;56(10):1042-1055.
15. Biggs BK, Rodgers KV, Nayman SJ, et al. Translation of a family-based behavioral intervention for adolescent obesity using the RE-AIM framework and common steps from adaptation frameworks. *Translational behavioral medicine* 2023;13(9):700-709.
16. James KS, Connelly CD, Rutkowski E, et al. Family-based weight management with latino mothers and children. *J. Spec. Pediatr. Nurs.* 2008;13(4):249-262.
17. Estabrooks PA, Shoup JA, Gattshall M, et al. Automated telephone counseling for parents of overweight children: a randomized controlled trial. *Am. J. Prev. Med.* 2009;36(1):35-42. e32.
18. Zoellner JM, You W, Hill JL, et al. A comparative effectiveness trial of two family-based childhood obesity treatment programs in a medically underserved region: Rationale, design & methods. *Contemp. Clin. Trials* 2019;84:105801.
19. Zoellner JM, You W, Hill JL, et al. Comparing two different family-based childhood obesity treatment programmes in a medically underserved region: Effectiveness, engagement and implementation outcomes from a randomized controlled trial. *Pediatric obesity* 2022;17(1):e12840.
20. Nowicka P, Höglund P, Pietrobelli A, et al. Family Weight School treatment: 1-year results in obese adolescents. *Int. J. Pediatr. Obes.* 2008;3(3):141-147.
21. Joosse L, Stearns M, Anderson H, et al. Fit Kids/Fit Families: a report on a countywide effort to promote healthy behaviors. *Wis. Med. J.* 2008;107(5):231.
22. Soltero EG, Konopken YP, Olson ML, et al. Preventing diabetes in obese Latino youth with prediabetes: a study protocol for a randomized controlled trial. *BMC Public Health* 2017;17(1):1-12.
23. Peña A, Olson ML, Hooker E, et al. Effects of a Diabetes Prevention Program on Type 2 Diabetes Risk Factors and Quality of Life Among Latino Youths With Prediabetes: A Randomized Clinical Trial. *JAMA network open* 2022;5(9):e2231196.
24. Orban K, Edberg A-K, Thorngren-Jerneck K, et al. Changes in parents' time use and its relationship to child obesity. *Phys. Occup. Ther. Pediatr.* 2014;34(1):44-61.
25. Önnerfält J, Erlandsson L-K, Orban K, et al. A family-based intervention targeting parents of preschool children with overweight and obesity: conceptual framework and study design of LOOPS-Lund overweight and obesity preschool study. *BMC Public Health* 2012;12(1):1-9.
26. Law C, Cole T, Cummins S, et al. A pragmatic evaluation of a family-based intervention for childhood overweight and obesity. *Public Health Res* 2014;2(5).
27. Sacher PM, Kolotourou M, Chadwick PM, et al. Randomized controlled trial of the MEND program: a family-based community intervention for childhood obesity. *Obesity* 2010;18(S1):S62-S68.
28. Wilson TA, Liu Y, Adolph AL, et al. Behavior modification of diet and parent feeding practices in a community-Vs primary care–centered intervention for childhood obesity. *J. Nutr. Educ. Behav.* 2019;51(2):150-161. e151.

29. Kitzman-Ulrich H, Wilson DK, St. George SM, et al. A preliminary test of a motivational and parenting weight loss program targeting low-income and minority adolescents. *Child Obes* 2011;7(5):379-384.
30. Chamay Weber C, Camparini N, Lanza L, et al. Parents' integration in the treatment of adolescents with obesity: A qualitative study. *Fam Syst Health* 2016;34(4):396.
31. Bocca G, Kuitert M, Sauer P, et al. A multidisciplinary intervention programme has positive effects on quality of life in overweight and obese preschool children. *Acta Paediatr.* 2014;103(9):962-967.
32. Bocca G, Kuitert MW, Sauer PJ, et al. Effect of a multidisciplinary treatment program on eating behavior in overweight and obese preschool children. *J. Pediatr. Endocrinol. Metab.* 2018;31(5):507-513.
33. Kitzman-Ulrich H, Hampson R, Wilson DK, et al. An adolescent weight-loss program integrating family variables reduces energy intake. *J Am Diet Assoc* 2009;109(3):491-496.
34. Ellis DA, Janisse H, Naar-King S, et al. The effects of multisystemic therapy on family support for weight loss among obese African-American adolescents: findings from a randomized controlled trial. *J. Dev. Behav. Pediatr.* 2010;31(6):461-468.
35. Idalski Carcone A, MacDonell KE, Naar-King S, et al. Treatment engagement in a weight loss intervention for African American adolescents and their families. *Child. Health Care* 2011;40(3):232-252.
36. Naar-King S, Ellis D, Kolmodin K, et al. A randomized pilot study of multisystemic therapy targeting obesity in African-American adolescents. *J. Adolesc. Health* 2009;45(4):417-419.
37. Flodmark C-E, Ohlsson T, Rydén O, et al. Prevention of progression to severe obesity in a group of obese schoolchildren treated with family therapy. *Pediatrics* 1993;91(5):880-884.
38. Ball GD, Mushquash AR, Keaschuk RA, et al. Using Intervention Mapping to develop the Parents as Agents of Change (PAC©) intervention for managing pediatric obesity. *BMC Res. Notes* 2017;10(1):1-11.
39. Ball GD, Ambler KA, Keaschuk RA, et al. Parents as Agents of Change (PAC) in pediatric weight management: The protocol for the PAC randomized clinical trial. *BMC Pediatr.* 2012;12(1):1-13.
40. Spence ND, Newton AS, Keaschuk RA, et al. Predictors of short-and long-term attrition from the parents as agents of change randomized controlled trial for managing pediatric obesity. *J. Pediatr. Health Care* 2017;31(3):293-301.
41. Spence ND, Newton AS, Keaschuk RA, et al. Parents as Agents of Change in Managing Pediatric Obesity: A Randomized Controlled Trial Comparing Cognitive Behavioral Therapy versus Psychoeducation Interventions. *Childhood obesity (Print)* 2023;19(2):71-87.
42. Steele RG, Aylward BS, Jensen CD, et al. Comparison of a family-based group intervention for youths with obesity to a brief individual family intervention: A practical clinical trial of positively fit. *J. Pediatr. Psychol.* 2011;37(1):53-63.
43. St. George SM, Wilson DK, Schneider EM, et al. Project SHINE: Effects of parent–adolescent communication on sedentary behavior in African American adolescents. *J. Pediatr. Psychol.* 2013;38(9):997-1009.
44. St. George SM, Wilson DK, Van Horn ML. Project SHINE: Effects of a randomized family-based health promotion program on the physical activity of African American parents. *J. Behav. Med.* 2018;41(4):537-549.
45. Nowicka P, Pietrobelli A, Flodmark C-E. Low-intensity family therapy intervention is useful in a clinical setting to treat obese and extremely obese children. *Int. J. Pediatr. Obes.* 2007;2(4):211-217.
46. Hadley W, McCullough MB, Rancourt D, et al. Shaking up the system: the role of change in maternal-adolescent communication quality and adolescent weight loss. *J. Pediatr. Psychol.* 2015;40(1):121-131.
47. Jelalian E, Hadley W, Sato A, et al. Adolescent weight control: An intervention targeting parent communication and modeling compared with minimal parental involvement. *J. Pediatr. Psychol.* 2015;40(2):203-213.
48. Herget S, Markert J, Petroff D, et al. Psychosocial well-being of adolescents before and after a 1-year telephone-based adiposity prevention study for families. *J. Adolesc. Health* 2015;57(3):351-354.
49. Markert J, Herget S, Petroff D, et al. Telephone-based adiposity prevention for families with overweight children (TAFF-Study): one year outcome of a randomized, controlled trial. *Int. J. Environ. Res. Public Health* 2014;11(10):10327-10344.

50. Markert J, Alff F, Zschaler S, et al. Prevention of childhood obesity: Recruiting strategies via local paediatricians and study protocol for a telephone-based counselling programme. *Obes. Res. Clin. Pract.* 2013;7(6):e476-e486.
